# Prognostic value and immune landscapes of anoikis-associated lncRNAs in lung adenocarcinoma

**DOI:** 10.18632/aging.205481

**Published:** 2024-02-05

**Authors:** Bo Wu, Xiang Zhang, Nan Feng, Zishun Guo, Lu Gao, Zhihua Wan, Wenxiong Zhang

**Affiliations:** 1 Department of Thoracic Surgery, The Second Affiliated Hospital, Jiangxi Medical College, Nanchang University, Nanchang 330006, China; 2 Department of Thoracic Surgery, Baoding No.1 Central Hospital, Baoding 071000, China

**Keywords:** anoikis, lncRNAs, lung adenocarcinoma, overall survival, drug sensitivity

## Abstract

Background: Methods for predicting the outcome of lung adenocarcinoma (LUAD) in the clinic are limited. Anoikis is an important route to programmed cell death in LUAD, and the prognostic value of a model constructed with anoikis-related lncRNAs (ARlncRNAs) in LUAD is unclear.

Methods: Transcriptome and basic information for LUAD patients was obtained from the Cancer Genome Atlas. Coexpression and Cox regression analyses were utilized to identify prognostically significant ARlncRNAs and construct a prognostic signature. Furthermore, the signature was combined with clinical characteristics to create a nomogram. Finally, we performed principal component, enrichment, tumor mutation burden (TMB), tumor microenvironment (TME) and drug sensitivity analyses to evaluate the basic research and clinical merit of the signature.

Results: The prognostic signature developed with eleven ARlncRNAs can accurately predict that high-risk group patients have a worse prognosis, as proven by the receiver operating characteristic (ROC) curve (AUC: 0.718). Independent prognostic analyses indicated that the risk score is a significant independent prognostic element for LUAD (P<0.001). In the high-risk group, enrichment analysis demonstrated that glucose metabolism and DNA replication were the main enrichment pathways. TMB analysis indicated that the high-risk group had a high TMB (P<0.05). Drug sensitivity analyses can recognize drugs that are sensitive to different risk groups. Finally, 11 ARlncRNAs of this signature were verified by RT-qPCR analysis.

Conclusions: A novel prognostic signature developed with 11 ARlncRNAs can accurately predict the OS of LUAD patients and offer clinical guidance value for immunotherapy and chemotherapy treatment.

## INTRODUCTION

Lung cancer (LC) is a carcinoma with high incidence and mortality rates [1]. Lung adenocarcinoma (LUAD) is an essential component of the pathobiological types of LC and is extremely heterogeneous [2]. With the continuous improvement of LUAD diagnostic and treatment protocols in the past decade, the survival rate has improved; however, the OS for LUAD patients is still unsatisfactory [3]. Even worse, commonly used clinical assessment metrics (including TNM staging) cannot accurately predict LUAD prognosis. Presently, biomarkers are a sensitive indicator used to identify patient survival, and multibiomarker prognostic signatures have better predictive power than a single biomarker [4, 5]. Hence, determining reliable multibiomarker prognostic features that predict the prognosis of LUAD patients is extremely important.

**Figure 1:**
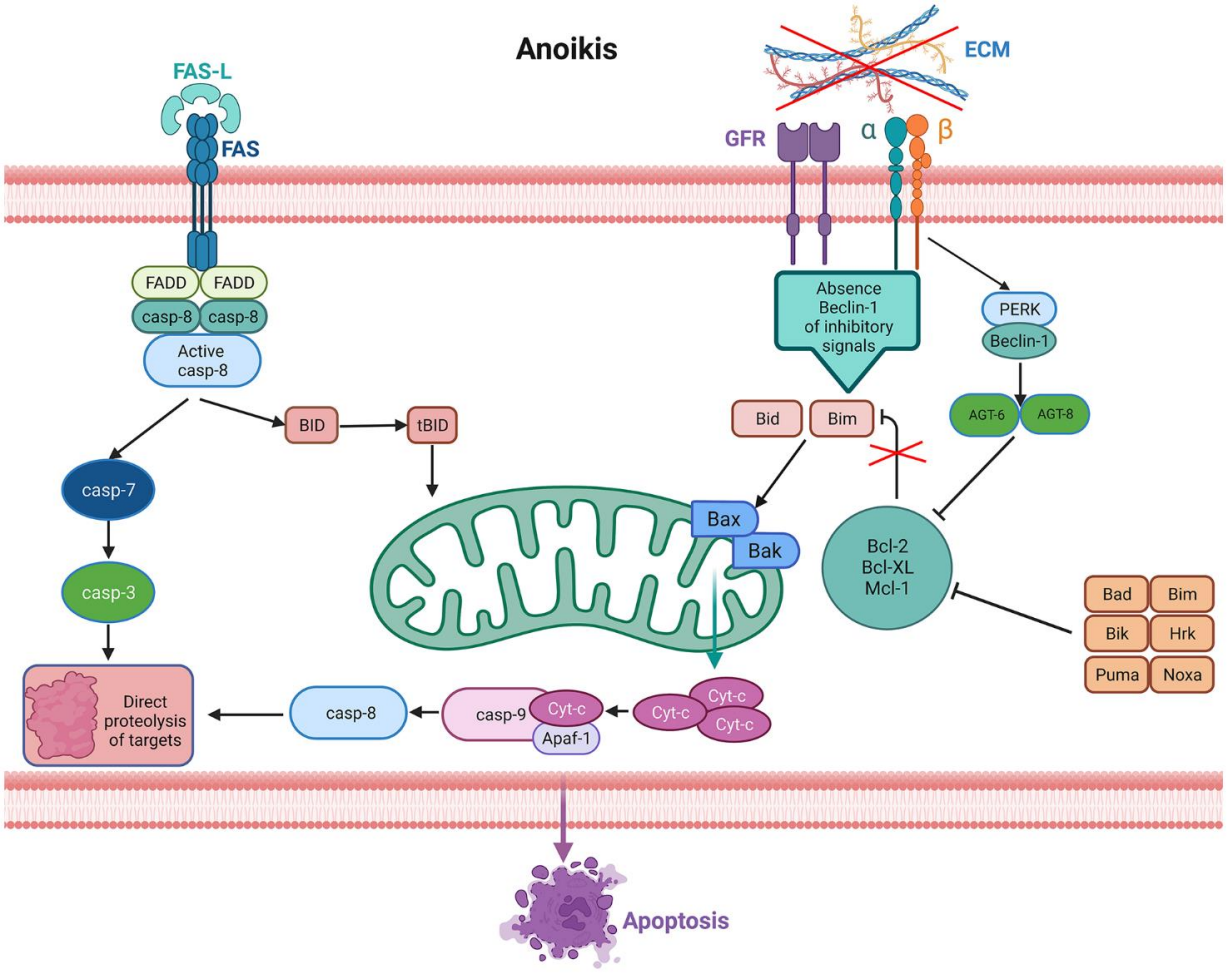
**Signaling pathways activated in anoikis.** Lack of the extracellular matrix (ECM) contact or improper ECM contact prevents the activation of pro-survival signals resulting in reduced anti-apoptotic pathways, thus activating anoikis from death receptors and mitochondria. Increased expression of extrinsic Fas receptors also activates the extrinsic pathway.

Programmed apoptosis is one of the major pathways of apoptosis in LUAD, in which anoikis plays an important role. Anoikis is a special type of programmed apoptosis that affects processes of cancer invasion and metastasis by disturbing mitochondria or automating cell surface death receptors, leading to apoptosis (Figure 1) [6, 7]. In recent years. Numerous studies have shown the discovery of anoikis as an important mechanism for cancer invasion and metastasis in gastric, breast, prostate and lung cancers [8–10]; for example, upregulation of the PDK4 gene leads to chemoresistance in LC and promotes cancer cell proliferation [10]. Despite the key role of anoikis genes in tumorigenesis and progression, the impact of anoikis on LUAD prognosis has rarely been studied. Moreover, long noncoding RNAs (lncRNAs) are engaged in cancer cell proliferation, migration and invasion processes [11]. Interestingly, lncRNAs have been reported to be closely correlated with anoikis in tumor cells. For example, the anti-apoptotic effect of MRPL23-AS1 in cystic carcinoma of the salivary gland was observed *in vitro* [12]. In ovarian cancer, lncRNA HOTAIR regulates the anti-apoptotic ability of neoplastic cells by influencing EZH2 [13]. The above studies indicate that anoikis-related lncRNAs (ARlncRNAs) have an essential effect on the progression of cancer. To date, no research on ARlncRNAs in LUAD prognosis has been conducted. Therefore, it is necessary to investigate the association between ARlncRNAs and the OS of LUAD.

In the current study, we identified significantly prognostic ARlncRNAs for developing a novel prognostic signature in LUAD patients and then verified its predictive ability. Furthermore, we analyzed the sensitivity of different risk groups to common drugs, which offers clinical guidance value for immunotherapy and chemotherapy therapies.

## MATERIALS AND METHODS

### Data acquisition

**Figure 2:**
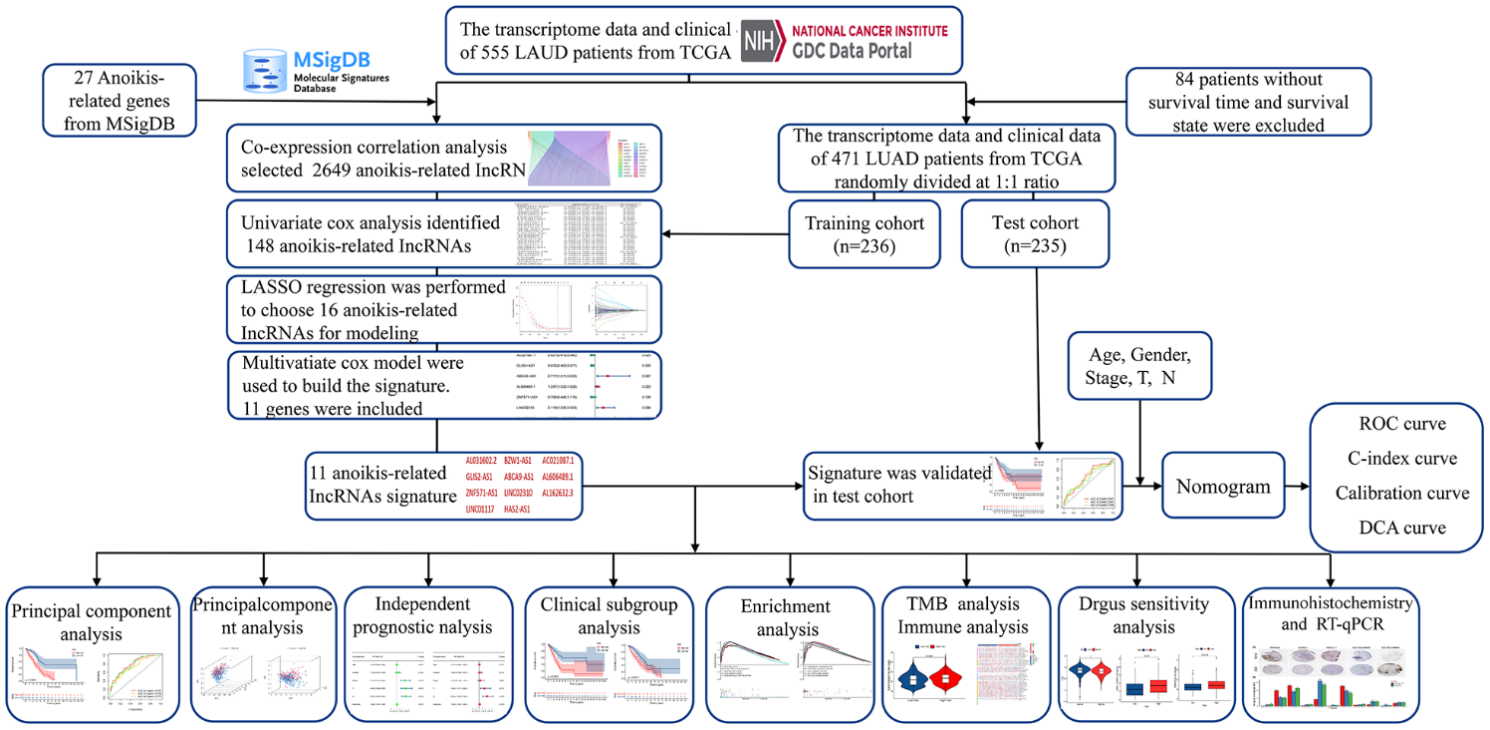
Overall flow diagram of the study.

Clinical information and gene expression messages of 555 (506 LUAD samples) samples were downloaded from The Cancer Genome Atlas (TCGA) on September 3, 2022 (https://portal.gdc.cancer.gov/). Thirty-five LUAD samples with no survival time, survival condition or complete gene information were further excluded, and 471 patients with full clinical information were enrolled in the TCGA-LUAD cohort. Then, the cohort was randomly assigned to training (236 samples) and test (235 samples) cohorts at a ratio of 1:1 (Table 1). Construct a prognostic signature using the most valuable ARlncRNAs filtered from the training cohort data. TCGA-LUAD and test cohorts were utilized to test the predictability and clinical applicability of the signature. The data processing flow is detailed in Figure 2. Statistical analyses were performed with R software (version 4.2.3).

**Table 1:** Clinicopathological characteristics of 471 LUAD patients in the TCGA database.

**Characteristic**	**Total cohort**	**Training cohort**	**Test cohort**	**P-value**
**Total**	471 (100%)	235 (50%)	236 (50%)	
**Age**				1.000
≤65	225 (47.77%)	111 (47.23%)	114 (48.31%)	
>65	236 (50.11%)	117 (49.79%)	119 (50.42%)	
Unknow	10 (2.12%)	7 (2.98%)	3 (1.27%)	
**Gender**				0.973
Female	256 (54.35%)	129 (54.89%)	127 (53.81%)	
Male	215 (45.65%)	106 (45.11%)	109 (46.19%)	
**Stage**				1.000
I	255 (54.14%)	129 (54.89%)	126 (53.39%)	
II	108 (22.93%)	52 (22.13%)	56 (23.73%)	
III	75 (15.92%)	35 (14.89%)	40 (16.95%)	
IV	25 (5.31%)	13 (5.53%)	12 (5.08%)	
Unknow	8 (1.70%)	6 (2.55%)	2 (0.85%)	
**T stage**				1.000
T1	160 (33.97%)	82 (34.89%)	78 (33.05%)	
T2	250 (53.08%)	122 (51.91%)	128 (54.24%)	
T3	39 (8.28%)	21 (8.94%)	18 (7.63%)	
T4	19 (4.03%)	7 (2.98%)	12 (5.08%)	
Unknow	3 (0.64%)	3 (1.28%)	0 (0.00%)	
**N stage**				1.000
N0	304 (64.54%)	147 (62.55%)	157 (66.53%)	
N1	87 (18.47%)	44 (18.72%)	43 (18.22%)	
N2	66 (14.01%)	31 (13.19%)	35 (14.83%)	
N3	2 (0.42%)	2 (0.85%)	0 (0.00%)	
Unknow	12 (2.55%)	11 (4.68%)	1 (0.42%)	
**M stage**				0.989
M0	318 (67.52%)	156 (66.38%)	162 (68.64%)	
M1	24 (5.10%)	12 (5.11%)	12 (5.08%)	
Unknow	129 (27.39%)	67 (28.51%)	62 (26.27%)	

Abbreviation: LUAD, Lung adenocarcinoma; TCGA, The Cancer Genome Atlas.

### Identification of ARlncRNAs

Twenty-seven anoikis-related genes were acquired from the Molecular Signatures Database (MSigDB) [14]. Coexpression analysis in the “limma” R package was used to screen potential ARlncRNAs between anoikis-related genes and lncRNAs (|R| >0.4; P < 0.001).

### Development of the ARlncRNA prognostic signature

Combining significant ARlncRNAs with the clinical data of LUAD patients in the training group, univariate Cox regression analysis was undertaken to identify ARlncRNAs correlated with OS. Next, least absolute shrinkage and selection operator (LASSO) and multivariate Cox regression were utilized to recognize statistically significant prognostic ARlncRNAs. Finally, we constructed a novel prognostic signature developed with the above prognostic ARlncRNAs by multivariate Cox regression and calculated risk scores per patient. Risk Score = (Exp lncRNA1 × β lncRNA1) + (Exp lncRNA2 × β lncRNA2) + (Exp lncRNA3 × β lncRNA3) + ...... + (Exp lncRNAn × β lncRNAn). The Exp represents was the expression of lncRNA and the β was the multivariate Cox regression analysis coefficient of lncRNA [15].

### Evaluating the ARlncRNA prognostic signature and principal component analysis (PCA)

Patients were assigned to high- and low-risk cohorts according to the risk score. Survival status maps and ARlncRNA expression heatmaps of patients from the two risk groups were mapped with the “pheatmap” R package. Kaplan-Meier curves were generated to compare the OS of the two risk groups with the “survival” R package. In addition, a receiver operating characteristic (ROC) curve was generated by the “timeROC” R package to assess the 1-, 3- and 5-year predictive capability of the prognostic signature and calculate the area under the curve (AUC). Finally, PCA was applied to estimate the capability of distinguishing between high- and low-risk patients via the “scatterplot3” and “limma” R packages [16].

### Independent prognostic analysis

We investigated the prognostic value of the risk score and clinical characteristics in LUAD patients via univariate Cox regression. Meanwhile, correlation analysis was utilized for risk scores and the above clinical characteristics. The predictive accuracy of this signature was verified via the C-index and ROC curves. Then, a new nomogram model was developed with clinical characteristics and risk scores. A calibration curve is utilized to show the agreement between the predicted and actual outcomes. Decision curve analysis (DCA) was utilized to demonstrate the clinical applicability value of this model.

### Clinical subgroup validation

The patients were assigned to different subgroups depending on clinicopathological characteristics in the training set. The subgroups were as follows: age (≤65; >65), sex (female; male), stage (stage I-II; stage III-IV), T stage (T1-2; T3-4), and N stage (N0; N1-3). The OS of patients in the high- and low-risk groups was compared via subgroup analysis, and the aim was to determine the optimal range of application of the signature.

### Gene set enrichment analysis (GSEA) and tumor mutation burden (TMB) analysis

Enrichment pathways for the two risk groups were examined by six different methods (kegg, go, reactome, biocarta, wikipathways, pid) in GSEA4.3.2 software, and |NES|>1 and FDR<0.25 were deemed reliable. TMB differences between the two risk groups were analyzed via the “maftools” R package [17]. Comparing the OS of patients in the high and low TMB groups via survival analysis.

### Tumor microenvironment (TME) analysis

We calculated the level of infiltration of important immune cells in two risk groups by seven methods (quentized, timer, epic, ciberspot, ciberspotabs, mcpcounter and xcell) [18], and the outcome was then represented by heatmaps. In addition, immune cell infiltration rates were compared between the two risk group populations by Wilcoxon analysis. Finally, based on the data calculated by the ciberspotabs method, correlation analysis was utilized to evaluate the level of immune cell infiltration in correlation with the risk score.

### Drug sensitivity analysis

Tumor immune dysfunction and exclusion (TIDE) data for LUAD were obtained via the website (http://tide.dfci.harvard.edu/) [19]. The relationship between risk scores and TIED was analyzed with the “ggpubr” and “limma” R packages (P < 0.05). Moreover, we analyzed the differentially expressed immune checkpoint genes among the two risk groups. Finally, analyses of the sensitivity to common drugs by the “pRRophetic” R package for both high- and low-risk groups (pFilter < 0.001 and corPvalue < 0.001).

### Immunohistochemistry staining and real-time quantitative polymerase chain reaction (RT-qPCR)

Immunohistochemical outcomes of differentially expressed anoikis-related genes between normal and tumor specimens were accessed through the Human Protein Atlas (HPA) database (https://www.proteinatlas.org/). In our study, human normal bronchial epithelium cells (BEAS-2B) and LUAD cells (NCI-H1395, NCI-H1975) were purchased from the Cell Bank of the Chinese Academy of Sciences (Shanghai, China). RNA was obtained from tissues with TRIzol reagent (Invitrogen, USA). HiScript II (Vazyme, China) was utilized to synthesize cDNA by reverse transcription. Primers for RT-qPCR experiments of 11 ARlncRNAs are shown in Supplementary Table 1. β-actin was used as an internal reference. The expression levels of lncRNAs were calculated using 2^ΔΔCT^.

### Availability of data and materials

The data sets used and/or analyzed during the current study are available from the corresponding author on reasonable request.

### Consent for publication

All authors gave their consent for publication.

## RESULTS

### Identification of ARlncRNAs and development of the prognostic signature

**Figure 3:**
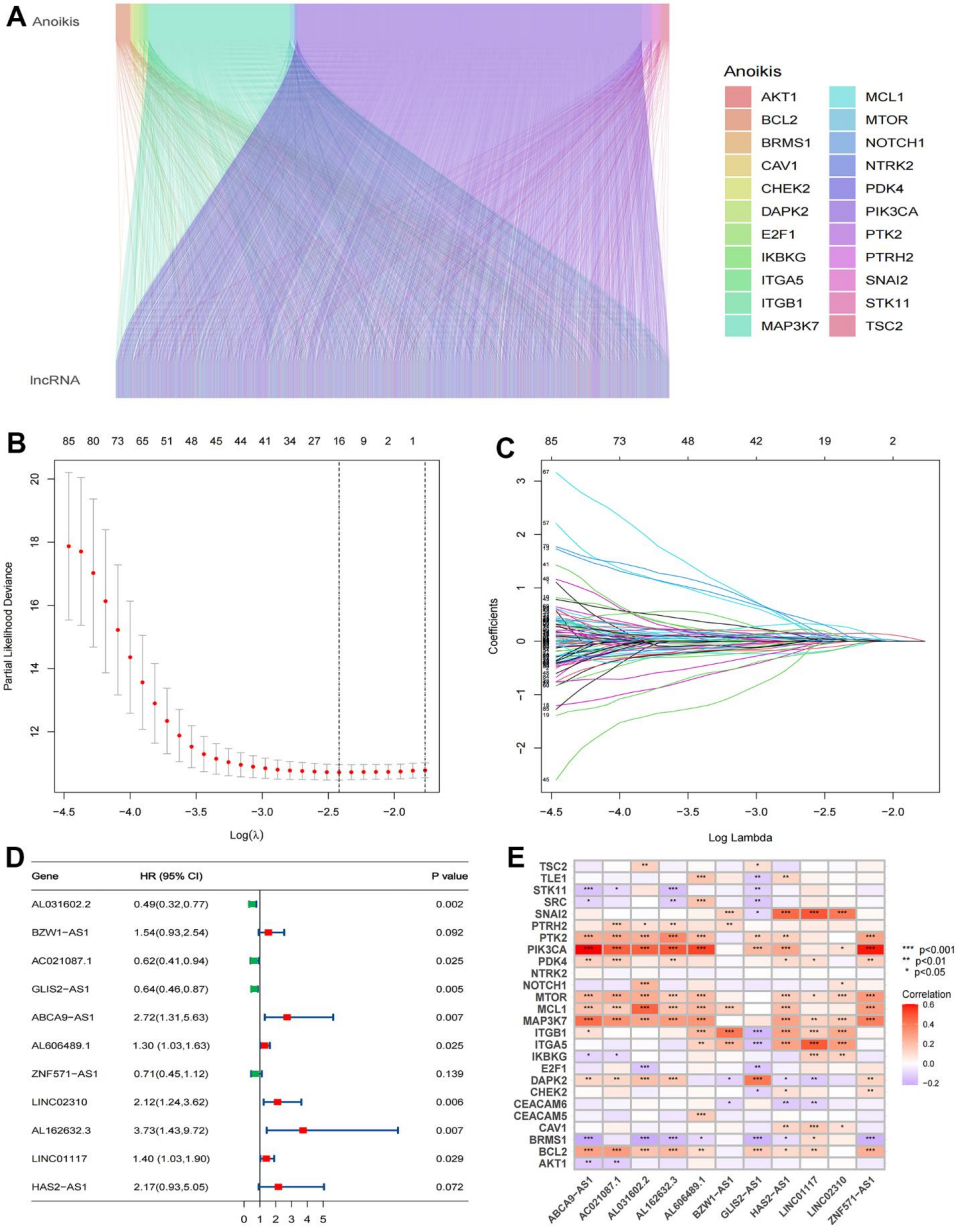
**Identification of significant prognosis ARlncRNAs.** (**A**) Sankey diagram depicting the relationships between 27 anoikis-related genes and ARlncRNAs co-expression. (**B**) LASSO Cox regression analysis revealed 85 ARlncRNAs based LASSO cross validation plot. (**C**) LASSO coefficient of 85 ARlncRNAs. (**D**) Forest plot showed different ARlncRNAs for high and low risk, with red representing high-risk lncRNAs and green representing low-risk ARlncRNAs. (**E**) Correlation heatmap showed the relationship between 11 ARlncRNAs and anoikis-related genes for the signature. Red represents positive correlations and blue represents negative correlations.

The analysis process of this research is displayed in a flow chart (Figure 2). First, 2649 ARlncRNAs were screened by coexpression analysis of 16,876 lncRNAs associated with 27 anoikis-related genes. Then, coexpression analysis results between anoikis-related genes and ARlncRNAs expressed by a Sankey diagram were obtained (Figure 3A). Next, combining the clinical data of patients in the training cohort, 148 ARlncRNAs associated with OS were identified among 2678 ARlncRNAs by univariate Cox regression analysis (Supplementary Table 2). Furthermore, 16 ARlncRNAs were significantly associated with prognosis from the above lncRNAs using LASSO regression analysis (Figure 3B, 3C). Finally, we identified 11 significant prognostic ARlncRNAs in LUAD patients via multivariate Cox regression analysis (Figure 3D). A correlation heatmap showed the relationship between 11 ARlncRNAs and anoikis-related genes (Figure 3E). The multivariate Cox regression analysis coefficient of ARlncRNAs is shown in Supplementary Table 3. Then, the risk scores of different patients will be counted according to the risk score formula, risk score = (Exp AL031602.2 × -0.71045017820589) + (Exp BZW1-AS1 × 0.432038348321673) + (Exp AC021087.1 × -0.476411291926875) + (Exp GLIS2-AS1 × -0.454008732783369) + (Exp ABCA9-AS1 × 0.999706491359515) + (Exp AL606489.1 × 0.259835252523394) + (Exp ZNF571-AS1 × -0.34874860401809) + (Exp LINC02310 × 0.75089202544965) + (Exp AL162632.3 × 1.31557357109109) + (Exp LINC01117 × 0.338762422003907) + (Exp HAS2-AS1 × 0.775846321855369). The Exp was the expression of lncRNA in different patients. According to the median risk score, patients were divided into the high-risk and the low-risk groups. The results of the risk scores and risk groups for each patient in the total, training and test cohorts are shown in Supplementary Tables 4–6.

### Evaluating the ARlncRNA prognostic signature and PCA

**Figure 4:**
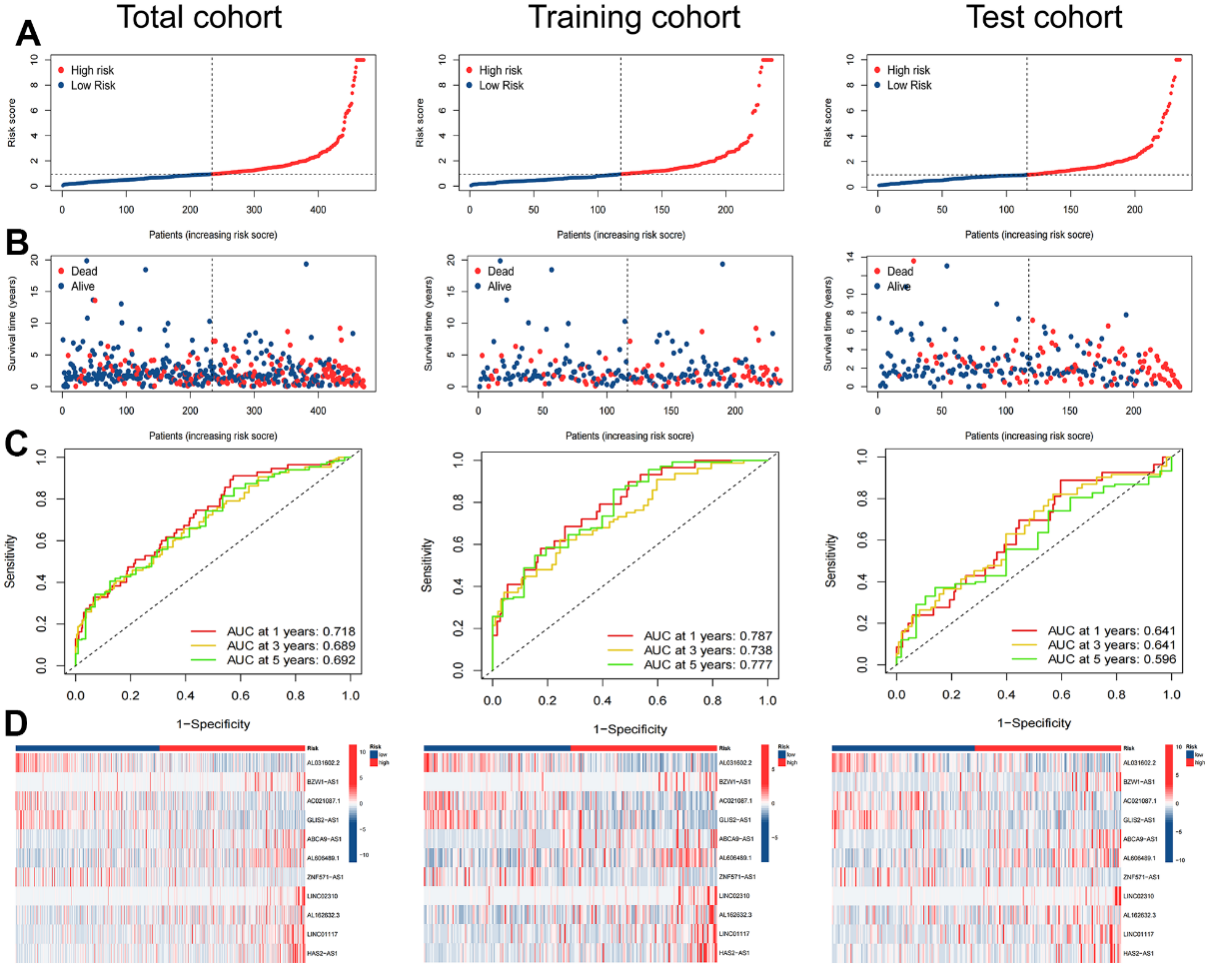
**Evaluation of the ARlncRNA prognostic signature in the total, training and test cohorts.** (**A**) Risk score distribution of patients with LUAD based on ARlncRNAs. (**B**) Scatter plots showed the association between the overall survival and the risk score distribution. (**C**) 1-, 3-, and 5-years overall survival area under the ROC curve of the signature. (**D**) Heatmap represented the expression of 11 ARlncRNAs involved in the signature.

**Figure 5:**
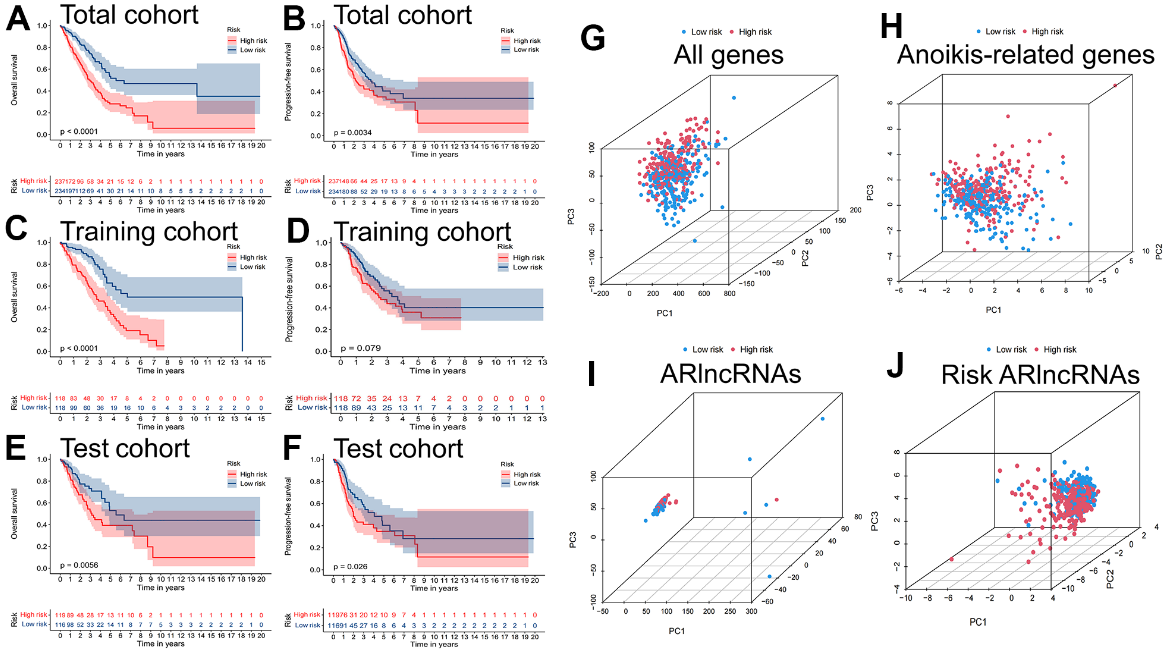
**Survival and PCA analysis of the prognostic signature.** Kaplan-Meier curves to compare the overall survival and progression-free survival of high-risk group and low-risk group in the (**A**, **B**) total, (**C**, **D**) training and (**E**, **F**) test cohort. Principal component analysis (PCA) based on (**G**) All genes, (**H**) Anoikis-related genes, (**I**) ARlncRNAs, and (**J**) Risk ARlncRNAs.

The TCGA-LUAD cohort was randomly assigned to training (236 samples) and test (235 samples) cohorts at a ratio of 1:1. We assigned the patients in each cohort to high- and low-risk cohorts according to the median risk score (Table 2), and the relationship between patients’ risk scores and groups is shown by risk curves (Figure 4A). The risk survival status plot shows the poor survival of high-risk LUAD patients (Figure 4B). The AUCs for 1-, 3-, and 5-year OS were 0.718, 0.689, and 0.692 (entire cohort); 0.787, 0.738, and 0.777 (training cohort); and 0.641, 0.641, and 0.596 (test cohort, Figure 4C). Furthermore, a heatmap of the expression profile of 11 ARlncRNAs in the signature demonstrated that ARlncRNAs AL031602.2, AC021087.1, GLIS2-AS1, and ZNF571-AS1 were highly expressed in the low-risk group. Conversely, BZW1-AS1, ABCA9-AS1, AL606489.1, LINC02310, AL162632.3, LINC01117, and HAS2-AS1 were expressed at low levels (Figure 4D). Survival analysis revealed that high-risk group patients were correlated with worse OS and progression-free survival in all three cohorts (Figure 5A–5F). Finally, PCA results indicated that all genes, anoikis-related genes, and ARlncRNAs were unable to validly recognize high- and low-risk patients, and only risk ARlncRNAs could effectively recognize high- and low-risk patients (Figure 5G–5J).

**Table 2:** Clinicopathological characteristics of LUAD patients in two risk groups.

**Characteristic**	**High-risk group**	**Low-risk group**
**Total**	**237 (50%)**	**234 (50%)**
**Age**		
≤65	111 (46.84%)	114 (48.72%)
>65	121 (51.05%)	115 (49.15%)
Unknow	5 (2.11%)	5 (2.14%)
**Gender**		
Female	115 (48.52%)	141 (60.26%)
Male	122 (51.48%)	93 (39.74%)
**Stage**		
I	104 (43.88%)	151 (64.53%)
II	66 (27.85%)	42 (17.95%)
III	45 (18.99%)	30 (12.82%)
IV	18 (7.59%)	7 (2.99%)
Unknow	4 (1.69%)	4 (1.71%)
**T stage**		
T1	59 (24.89%)	101 (43.16%)
T2	138 (58.23%)	112 (47.86%)
T3	28 (11.81%)	11 (4.70%)
T4	10 (4.22%)	9 (3.85%)
Unknow	2 (0.84%)	1 (0.43%)
**N stage**		
N0	133 (56.12%)	171 (73.08%)
N1	56 (23.63%)	31 (13.25%)
N2	41 (17.30%)	25 (10.68%)
N3	0 (0.00%)	2 (0.85%)
Unknow	7 (2.95%)	5 (2.14%)
**M stage**		
M0	161 (67.93%)	157 (67.09%)
M1	17 (7.17%)	7 (2.99%)
Unknow	59 (24.89%)	70 (29.91%)

Abbreviation: LUAD, Lung adenocarcinoma.

### Independent prognostic analysis

**Figure 6:**
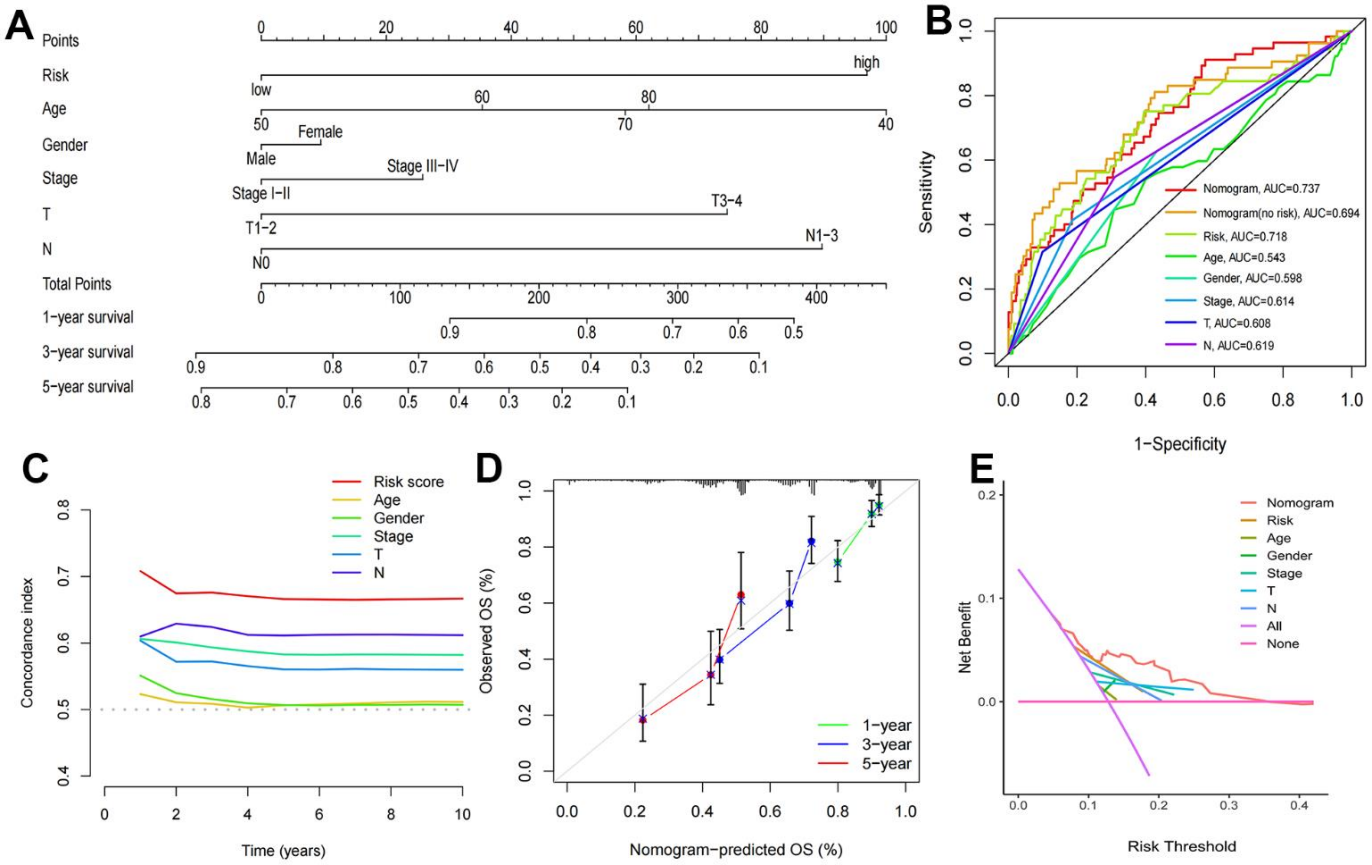
**Construction of nomogram and validation of its predictive ability.** (**A**) The nomogram for predicting the overall survival of patients with LUAD at 1-, 3-, and 5- years. (**B**) ROC curves for the risk score and other clinical characteristics to predict the overall survival rates of patients with LUAD. (**C**) C-Index curve for the risk score and other clinical characteristics. (**D**) The calibration curve for evaluating the nomogram. (**E**) DCA curve of the nomogram.

Cox regression analysis indicated that T stage (P=0.010), N stage and risk score (P<0.001) were independent prognostic factors (Table 3 and Supplementary Figure 1A, 1B). In addition, correlation analysis revealed that stage (P=0.0019), sex (P=0.001), T stage (P=0.00072), and N stage (P<0.0001) had significant relationships with risk scores, while age (P=0.88) was not significantly related to risk scores (Supplementary Figure 1C–1G). Finally, we developed a new nomogram with risk scores and clinical characteristics for predicting the OS of LUAD patients at 1, 3 and 5 years (Figure 6A). The ROC curve results showed that the risk score (AUC: 0.718) had better predictive ability than age (0.543), sex (0.598), stage (0.614), T stage (0.608) and N stage (0.619) (Figure 6B), which was also indicated by the C-index curves (Figure 6C). The nomogram with risk score had more accurate predictive prognostic ability compared to no risk scores (AUC: 0.737 vs. 0.694, Figure 6B), which precisely forecasted the OS of patients in the test cohort (Supplementary Figure 2). Calibration curves show the agreement between the predicted and actual outcomes, and DCA demonstrated the clinical applicability value of this model (Figure 6D, 6E).

**Table 3:** Univariate and multivariate Cox regression analysis based on risk factors (Training cohort).

**Characteristic**	**Univariate analysis**				**Multivariate analysis**		
	**HR**	**HR (95% CI)**	**P-value**		**HR**	**HR (95% CI)**	**P-value**
**Age**	1.01	(0.99, 1.03)	0.227		1.010	(1.00, 1.03)	0.173
**Gender**	1.16	(0.85, 1.56)	0.350		1.080	(0.79, 1.47)	0.618
**Stage**	2.31	(1.67, 3.20)	<0.001		1.160	(0.75, 1.79)	0.498
**T**	2.44	(1.66, 3.58)	<0.001		1.800	(1.15, 2.82)	0.010
**N**	2.47	(1.82, 3.35)	<0.001		2.220	(1.55, 3.20	<0.001
**Risk Score**	1.05	(1.03, 1.06)	<0.001		1.050	(1.04, 1.07)	<0.001

Abbreviations: CI, Confidence interval; HR, Hazard ratio.

### Clinical subgroup validation

The heatmap shows the expression of 11 ARlncRNAs in different clinical subgroups (Supplementary Figure 3A). To determine the optimal range of application of this signature, survival analysis was performed for the two risk groups in the subgroups. Kaplan-Meier curves showed that the high-risk group was related to worse OS in all subgroups (Supplementary Figure 4B–4F). Moreover, this difference was more significant in the age ≤ 65, stage I-II, T1-2 and N0 subgroups (P<0.001).

### GSEA and TMB analysis

**Figure 7:**
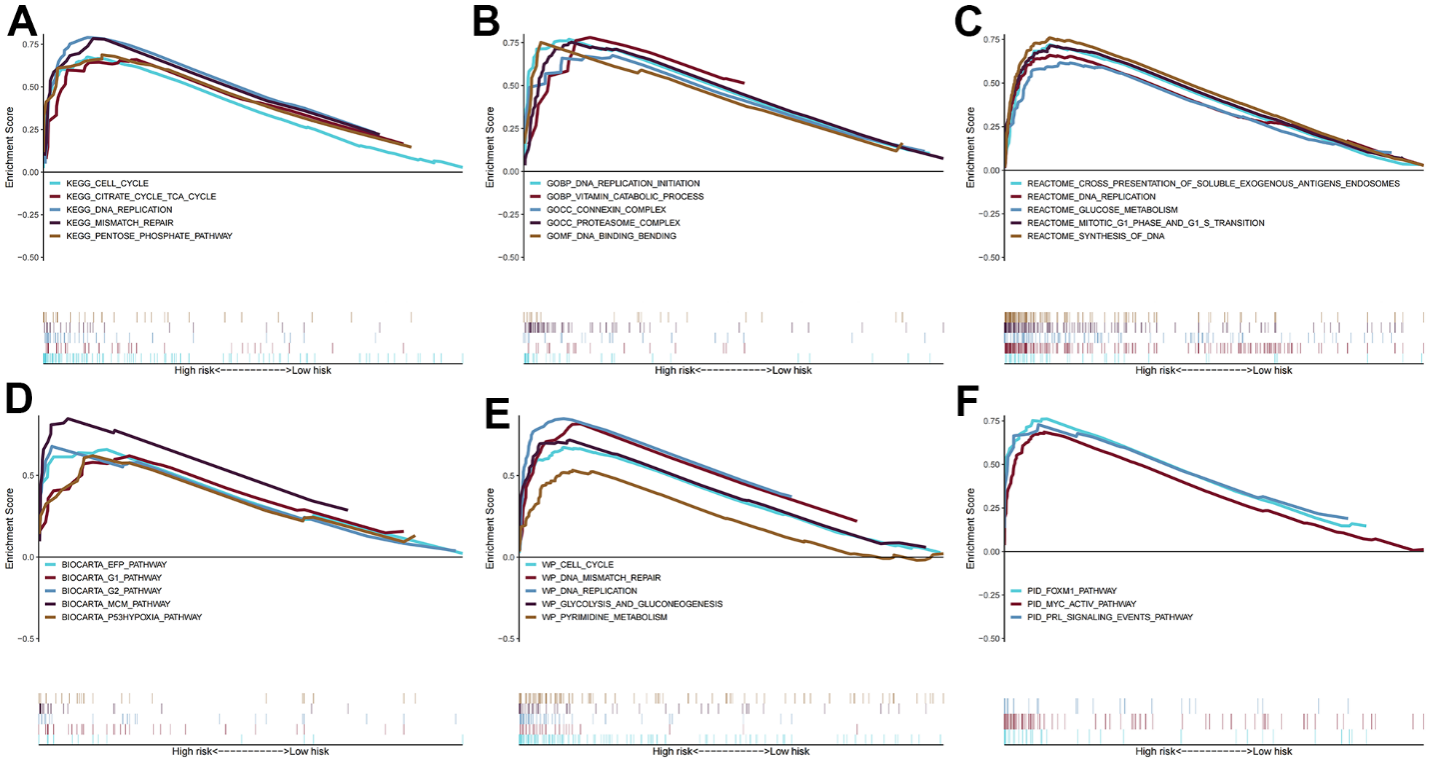
**Gene set enrichment analysis.** The pathways of (**A**) KEGG, (**B**) GO, (**C**) BIOCARTA, (**D**) REACTOME, (**E**) WIKIPATHWAYS, and (**F**) PID enriched in the low- and high- risk group.

**Figure 8:**
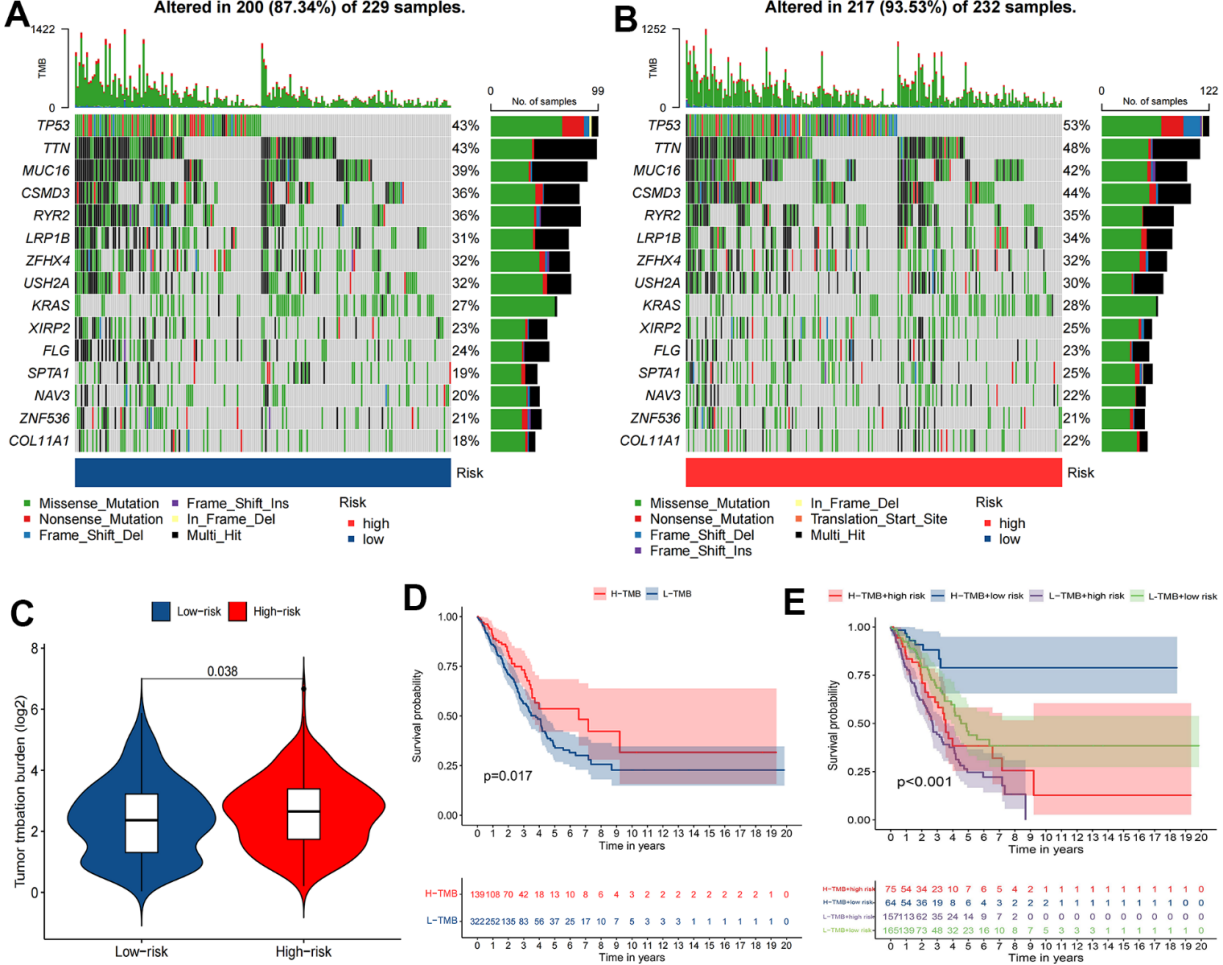
**The relationship between TMB and the signature.** (**A**, **B**) Waterfall plot revealed the top 15 mutation genes in LUAD for the two risk groups. (**C**) Differential TMB between two risk groups in LUAD. (**D**, **E**) Kaplan-Meier survival curves for the high and low TMB groups and a combined TMB-risk survival curve.

Enrichment results for six different pathway bases showed that glucose metabolism and DNA replication were the main enrichment pathways in the high-risk group. Notably, there was no significant enrichment pathway in the low-risk group (Figure 7). Depending on the results of the maftools algorithm, waterfall plots show that the mutation frequency of most genes was markedly increased in the high-risk group, such as TP53, TTN, MUC16, etc. (Figure 8A, 8B). Furthermore, the high-risk group was significantly linked to a high TMB. (Figure 8C). Survival analysis based on TMB results showed that the high TMB group had good OS compared to the low TMB group (Figure 8D, 8E).

### Tumor microenvironment analysis

**Figure 9:**
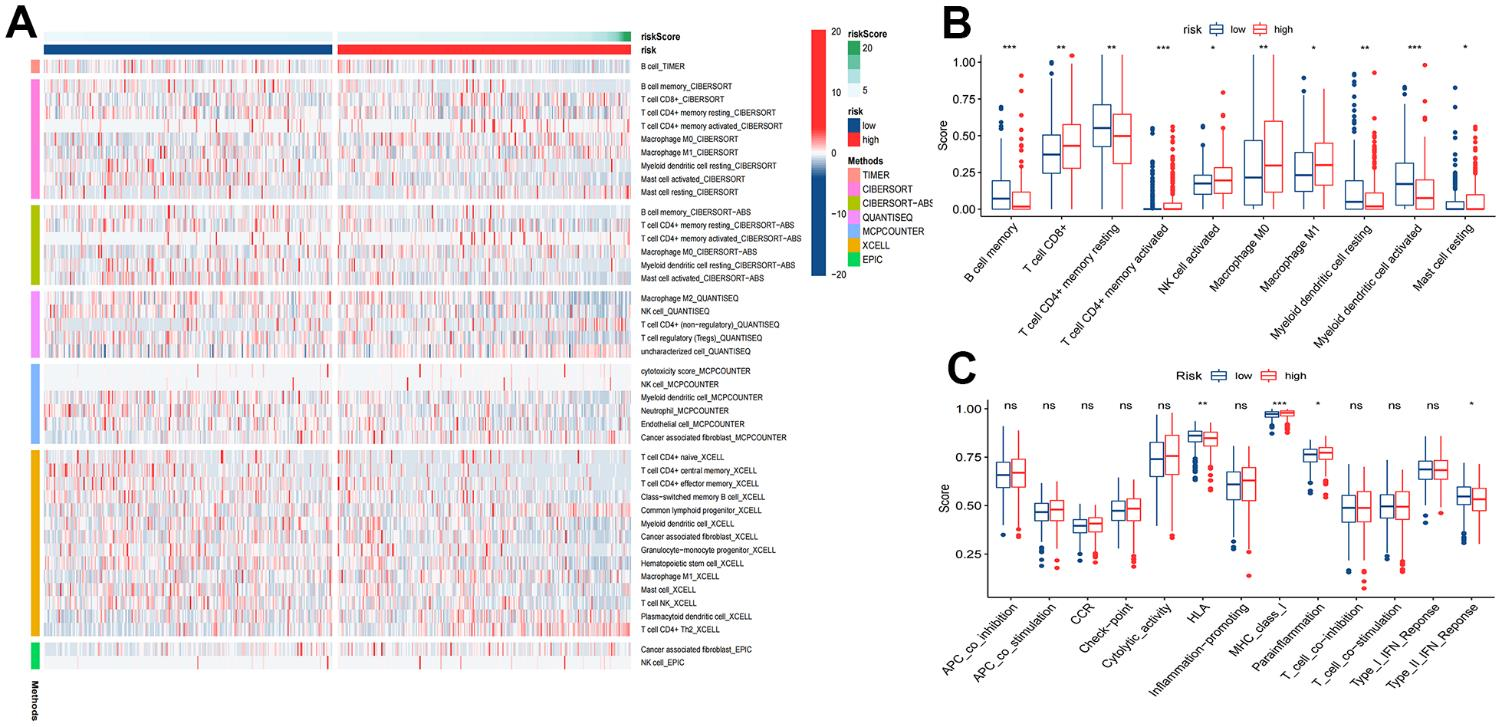
**Tumor immune microenvironment analysis.** (**A**) Heatmap for immune responses based on TIMER, CIBERSORT, CIBERSORT-ABS, QUANTISEQ, MCPCOUNTER, XCELL, and EPIC algorithms among high and low risk groups. (**B**) ssGSEA algorithm shows immune cells scores between the two risk groups. (**C**) ssGSEA algorithm shows immune functions scores between the two risk groups. *P<0.05; **P<0.01; ***P<0.001.

Most immune cell infiltration levels were noticeably dissimilar in the two risk groups, as shown by the heatmap (Figure 9A). Wilcoxon analysis showed significantly lower infiltration rates of activated myeloid dendritic cells and B-cell memory in the high-risk group. In contrast, the infiltration rates of M0 macrophages and CD8+ T cells were significantly higher (Figure 9B). Moreover, immune function analysis demonstrated that 4 (HLA, MHC class I, parainflammation, type II IFN response) immune function pathways were significantly different between the two risk groups (Figure 9C).

### Immunotherapy and chemotherapy sensitivity

**Figure 10:**
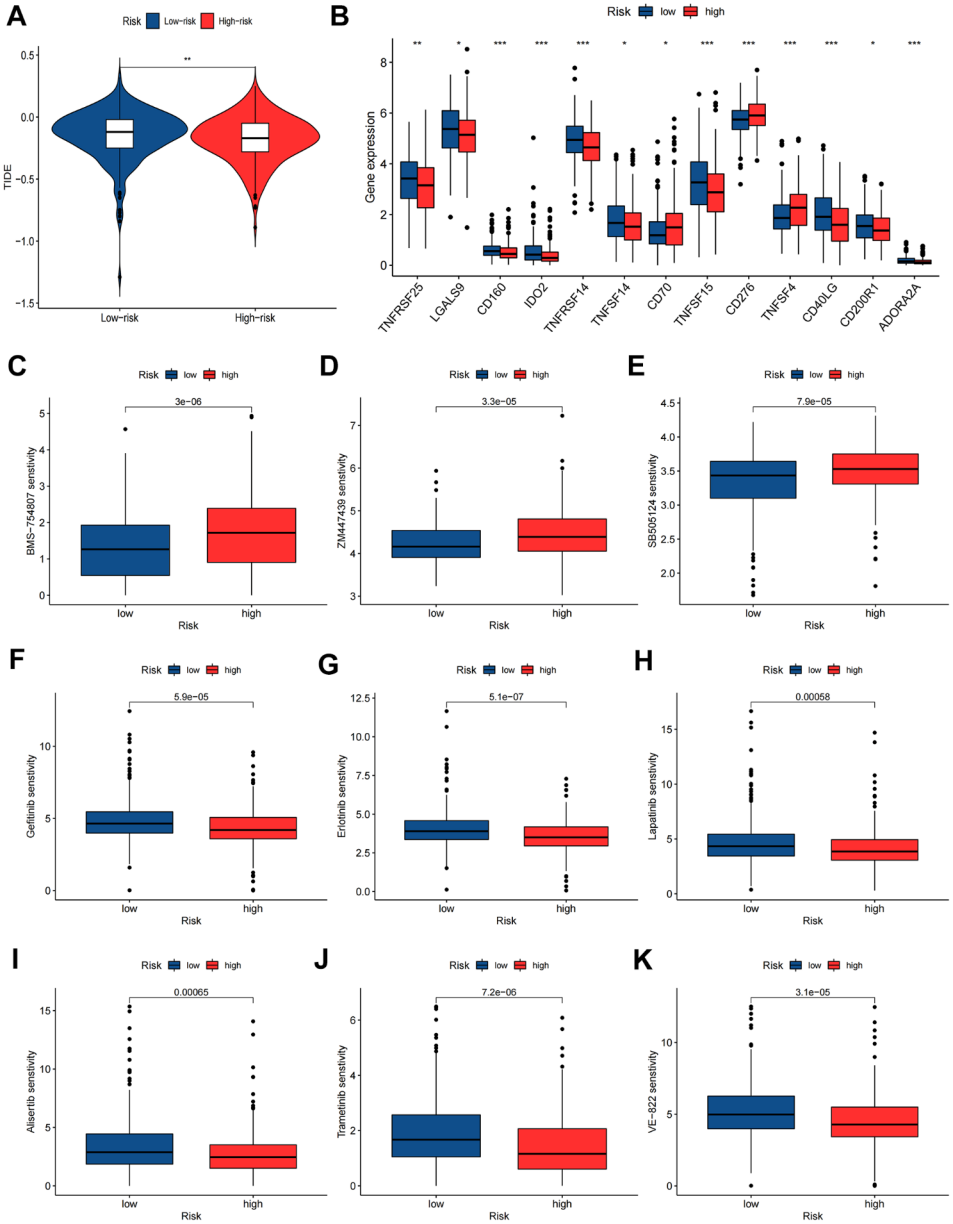
**Immunotherapy and chemotherapy sensitivity.** (**A**) Tumor immune dysfunction and exclusion (TIDE) algorithm analysis for the high-risk and low-risk groups. (**B**) Immune checkpoint genes expression level between high-risk and low-risk groups. (**C**–**K**) Chemosensitivity difference between high-risk and low-risk groups. *P<0.05; **P<0.01; ***P<0.001.

According to the results of the TIED algorithm, tumor cells in the low-risk group appeared more easily to be immune escapees (Figure 10A). Gene expression analyses of immune checkpoints indicated that the highly expressed immune checkpoint genes were TNFRSF25, LGALS9, CD160, IDO2, TNFRSF14, TNFSF14, TNFSF15, CD40LG, CD200R1 and ADORA2A in the low-risk group and CD70, CD276 and TNFSF4 in the high-risk group (Figure 10B). Drug sensitivity analyses revealed that low-risk group patients had 6 sensitive drugs, and the pharmacological action of these drugs is mainly by interdicting the PI3K/MTOR and ERK MAPK signaling pathways (Supplementary Table 7). The high-risk group had 59 sensitive drugs, and the pharmacological effect was mainly mediated by blocking mitosis and the IGF1R signaling pathway (Supplementary Table 8 and Figure 10C–10K).

### Immunohistochemistry staining and RT-qPCR

Protein expression maps of differentially expressed anoikis-associated genes in the HPA database showed that PDK4, CAV1, and MCL1 were expressed at relatively low levels in LUAD. In contrast, CEACAM5 and CEACAM6 were relatively highly expressed (Supplementary Figure 4A). Primers for RT-qPCR experiments of 11 ARlncRNAs are shown in Supplementary Table 1. RT-qPCR measurements showed significant differential expression of the above 11 ARlncRNAs in human normal bronchial epithelium cells (BEAS-2B) and LUAD cells (Supplementary Figure 4B). These results are in good agreement with the outcome of our analyses in the TCGA database.

## DISCUSSION

LUAD is the most predominant pathobiological type of LC and is highly heterogeneous [1, 2]. With the continuous improvement of LUAD diagnostic and treatment protocols in the past decade, the OS for LUAD patients is still unsatisfactory [3]. Traditional TNM staging and clinical prediction models cannot effectively predict the OS of LUAD and guide therapy [5]. It is urgent to discover a new predictive model. anoikis is a special type of programmed apoptosis that occurs when cancer cells are separated from the extracellular matrix (ECM), which influences tumorigenesis, metastasis and invasion [9]. Recently, lncRNAs have been reported to be closely correlated with anoikis in tumor cells [20]. Therefore, exploring the effect of ARlncRNAs on the prognosis of LUAD is necessary. The study identified significantly prognostic ARlncRNAs to develop a novel prognostic signature that can validly predict the OS of LUAD. In the high-risk group, enrichment analysis demonstrated that glucose metabolism and DNA replication were the main enrichment pathways. Patients in the high-risk group were immunosuppressed and had a high TMB. Two risk groups with different sensitivities to immunotherapy and different chemotherapeutic drugs.

We developed a novel prognostic signature with accurate predictive ability based on 11 ARlncRNAs. Using lncRNAs to construct prognostic signatures is widely used in colorectal, esophageal and breast cancer [21]. The results of this study are similar to theirs. Poorer prognosis in the high-risk group and prognostic features accurately predict patient prognosis. To more accurately predict LUAD prognosis. We combined the signature with clinical characteristics to create a new nomogram, which has more accurate predictive ability than the nomogram without risk scores (AUC: 0.737 vs. 0.694). Since we incorporated risk scores with high predictive prognostic power, our model also has advantages over traditional TNM stage (AUC: 0.737 vs. 0.614), and has better predictive ability than other existing models [22, 23]. Two ARlncRNAs in the signature have been reported in the relevant literature. Zhao et al. demonstrated that LINC02310 significantly promoted the growth and proliferation of LUAD [24]. Experiments showed that HAS2-AS1 overexpression strongly reduced breast cancer cell viability, migration and invasion [25]. This is consistent with our study results. Mechanisms of action for other ARlncRNAs still need to be further explored. RT-qPCR also confirmed the significant differential expression of the above 11 ARlncRNAs in normal and tumor cells.

GSEA showed that glucose metabolism and DNA replication were the main enrichment pathways in the high-risk group. The EGFR signaling pathway enhances SCAP N-glycosylation to deactivate SREBP-1 by promoting glucose uptake, and SREBP-1 can promote the progressive metastasis of cancer [26]. The DNA replication process plays a vital role in cell division and cancer progression [27]. The TME is involved in tumorigenesis, growth and metastasis [28]. The immune hypothesis suggests that fewer immune cancer cells in immunocompetent hosts evade antitumor immune responses. This may lead to an increase in immunosuppressive cells and a decrease in immunoreactive cells [29]. The study’s immune infiltration analyses revealed that the high-risk group had higher NK cell infiltration but lower mast cell and helper T-cell infiltration than the low-risk group. This high level of immunosuppression and poor immune responsiveness may contribute to the poor prognosis of high-risk patients. Immune function analysis showed differences in HLA, MHC class I, parainflammatory, and type II IFN responses between the two risk groups. Previous studies have demonstrated that decreased expression of HLA protein on the surface of cancer cells is an important mechanism of immune escape of tumor cells [30]. Furthermore, TMB is usually employed as a predictive indicator for immunotherapy in urothelial, lung and head and neck squamous cell cancers [31]. Our study found that the top two significantly different mutated genes between the high and low risk groups were TP53 and TTN. TP53 is the most mutable tumor suppressor gene, and its mutation not only increases the chance of carcinogenesis but also decreases antitumor activity [32]. TTN is also involved in the progression of many cancers. Jia et al. found that TTN regulates CDK5 through miR-142-5p to promote migration and invasion in LUAD [33]. This study found that patients with high TMB have a better prognosis. Studies have shown that patients may benefit more from immunotherapy with higher TMB [34]. However, in lung cancer patients with EGFR mutations, a higher TMB is associated with a poorer response to targeted therapy [35]. Additionally, for surgically resected early-stage lung cancer patients, a higher TMB is correlated with longer survival, potentially allowing for the avoidance of postoperative adjuvant chemotherapy [36]. Our findings reconfirmed that the TMB affects the prognosis of patients with LUAD, which may provide guidance for immunotherapy, targeted therapy and chemotherapy.

TIDE plays an essential role in tumor progression and is an essential tool for predicting the effectiveness of immunotherapy in oncology. Studies have demonstrated that oncology patients with lower TIDE can benefit significantly from immunotherapy [37]. Our study found lower TIDE scores in the high-risk group, which suggests that these patients are more sensitive to immunotherapy. Thus, we further explored the distinction in immunotherapy response among the two risk groups of LUAD patients. The expression of immune checkpoint genes was analyzed, and the results showed 12 differentially expressed immune checkpoint genes, 6 of which were highly expressed in high-risk populations, and the rest were highly expressed in low-risk populations. These genes could be potential targets for immunotherapy [38]. Furthermore, we collected common chemotherapeutic drugs, and the sensitivities of the two risk groups to these drugs were analyzed via the pRRophetic algorithm [39]. Our results revealed that the sensitive drugs in the high-risk group of patients mainly blocked mitosis and the IGF1R signaling pathway but were resistant to the PI3K/MTOR and ERK MAPK signaling pathways. This indicated that this prognostic signature can predict chemotherapy drug sensitivity in LUAD patients. Overall, the ARlncRNA prognostic signature can offer clinical guidance value for immunotherapy and chemotherapy treatment.

The strengths of this study are as follows: This study is the first to analyze the relationship between ARlncRNAs and prognosis of LUAD patients and attempts to use ARlncRNAs as a biological prognosticator for predicting LUAD patients. Second, the model performed better in predicting patient prognosis compared with the traditional model, which indicates the potential clinical application of ARlncRNAs in LUAD prognosis prediction. Third, this study pioneered the use of six gene enrichment analysis methods and searched for common metabolic pathways, providing a deep understanding of the intrinsic mechanisms of LUAD. Fourth, immune function, immune checkpoint and TIED analysis revealed differences in the immune landscape between high and low risk groups, providing new ideas for future studies, and finally, validated using cell lines, which lends credibility to the study. Nonetheless, some limitations of this study should be mentioned. First, LUAD patients from a single data source TCGA database. Second, the molecular mechanism of ARlncRNAs in the OS of LUAD is unclear and will be explored in future studies.

## CONCLUSIONS

We developed a novel signature with 11 ARlncRNAs that can precisely predict the OS of LUAD patients. Nomograms with risk scores have more accurate predictive prognostic power than those without risk scores. The high-risk group had gene enrichment mainly in glucose metabolism and DNA replication pathways, low immune response and high TMB. The novel signature can provide guidance for clinical immunotherapy and chemotherapy treatment. Due to these shortcomings, our study requires basic experimentation to discover the molecular mechanisms of ARlncRNAs in LUAD, and the selection of relevant sensitive drugs must be confirmed by clinical use.

## Supplementary Material

Supplementary Figures

Supplementary Tables 1-3

Supplementary Table 4

Supplementary Table 5

Supplementary Table 6

Supplementary Tables 7 and 8
