# Supplementary material for: Prognostic value and immune landscapes of anoikis-associated lncRNAs in lung adenocarcinoma
Source: Aging (Albany NY). 2024 Feb 5;16(3):2273–98. doi: 10.18632/aging.205481 (PMC10911388; doi:10.18632/aging.205481)
Supplement: Supplementary Figures [file aging-16-205481-s001.pdf]

SUPPLEMENTARY FIGURES

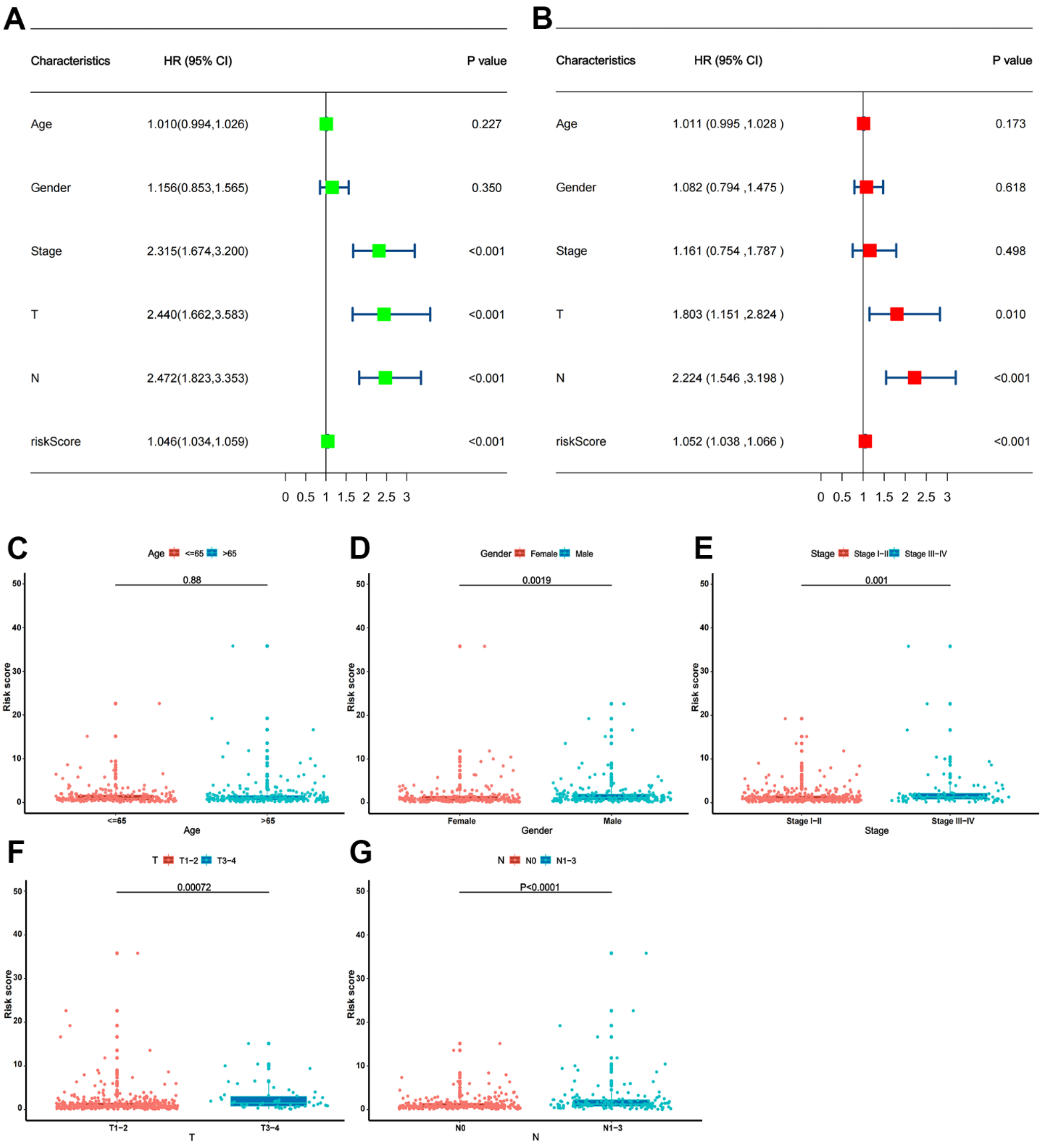

Supplementary Figure 1. Independent prognostic analysis of the prognostic signature. (**A**) Univariate and (**B**) Multivariate Cox regression analysis to examine the value of clinical characteristics and risk score as independent prognostic predictors. Correlation analysis of risk score with age (**C**), gender (**D**), stage (**E**), T stage (**F**) and N stage (**G**).

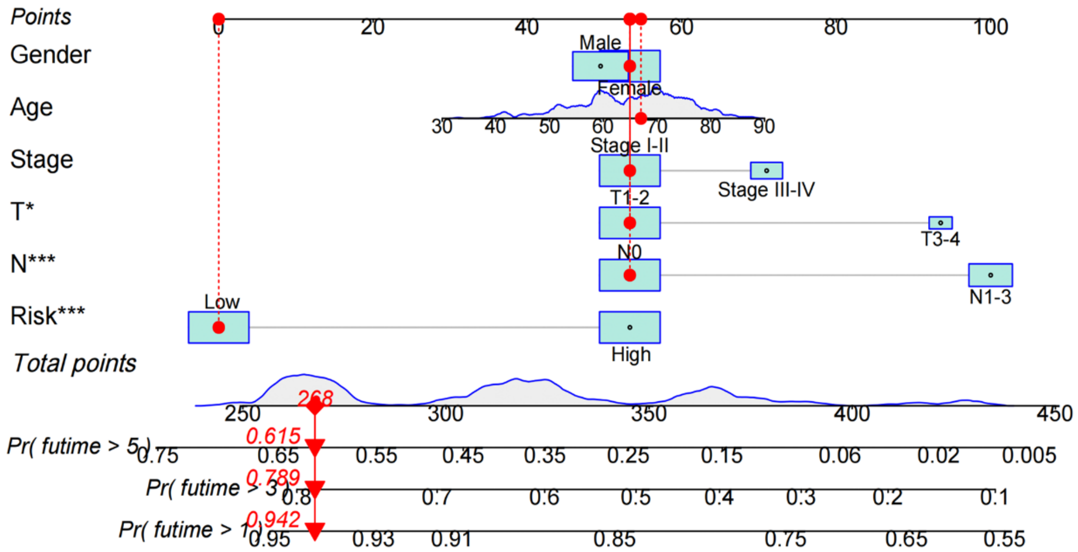

Supplementary Figure 2. Nomograms for predicting 1-, 3-, 5-year overall survival for a randomly selected patient in test cohort.

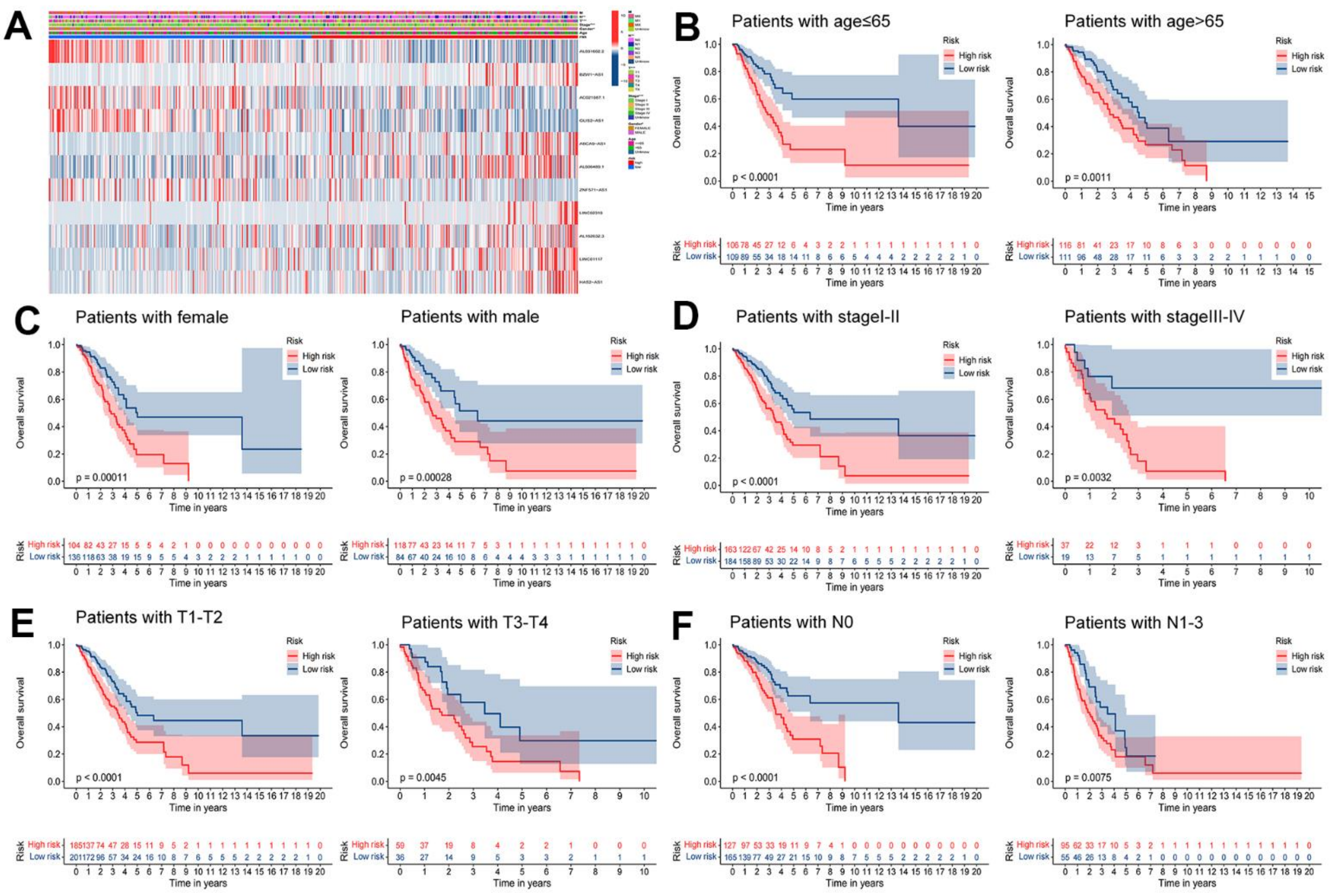

Supplementary Figure 3. Subgroup validation based on clinical features. (**A**) Heatmap for expression levels and correlation of risk signature with clinical factors. Kaplan-Meier survival analysis between high- and low-risk groups stratified by clinical characteristics: (**B**) age (≤65 and >65), (**C**) gender (female and male), (**D**) stage (I-II and III-IV), (**E**) T stage (T1-2 and T3-4) and (**F**) N stage (N0 and N1-3).

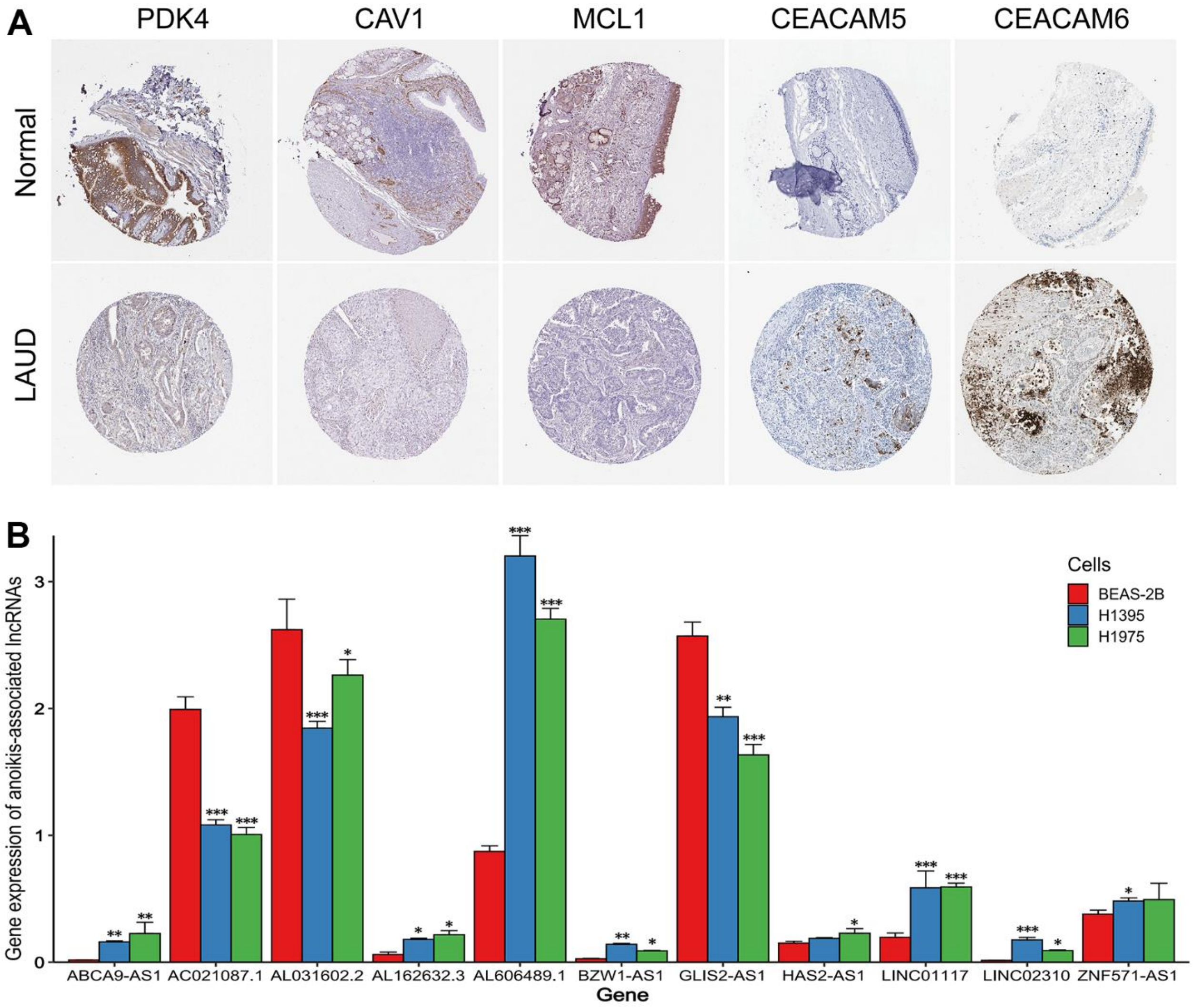

Supplementary Figure 4. Validation of apoptosis-related genes and ARlncRNAs expression levels. (**A**) Protein expression map of the top 5 differential apoptosis-related genes in normal and tumor tissues. (**B**) Expression levels of 11 ARlncRNAs in high and low risk groups.
