# Supplementary material for: Prognostic value and immune landscapes of anoikis-associated lncRNAs in lung adenocarcinoma
Source: Aging (Albany NY). 2024 Feb 5;16(3):2273–98. doi: 10.18632/aging.205481 (PMC10911388; doi:10.18632/aging.205481)
Supplement: Supplementary Tables 1-3 [file aging-16-205481-s002.pdf]

SUPPLEMENTARY TABLES

Supplementary Table 1. Primers for RT-qPCR experiments of 11 ARlncRNAs.

Gene	Forward primer	Reverse primer
AL031602.2	ATCTTCCCTGTCACCTCCCTT	CTGGGACATTCACACGGATG
BZW1-AS1	AGGACACAGGCCGAAGATTG	CACACTCGGGAGACAACCG
AC021087.1	CTCGAGCAGGGACGTATCAC	AAAGGGCAAGATAACGGCCA
GLIS2-AS1	TGCTAATTACTGCGGGGTCC	AAACTGAGGAACGGAAGGCTC
ABCA9-AS1	TCCAGTGTTCTGCACCAGTT	TGGCCCAACGTCTAATAGGG
AL606489.1	TCCTTTATCGGGCAAGAGGC	TGGGTGCAATGAGAGTGCAT
ZNF571-AS1	CGCCATCCCCTTTTCCTCTT	CGGTCCAAAGGTTCTCCGAA
LINC02310	GGGATAAATCCTCCCTGCCC	TCAGTTTTGCAGAAGGGGCT
AL162632.3	TGGCCGTAAAGTAAAACCCT	TCGGCCCAAAGGAGAAGAAA
LINC01117	CCCCGTGCTCGTTACAATCT	CTCCGGGGTTACTGGAGTCT
HAS2-AS1	CCCGGTTCTCACCCTCATAC	GAATTCGTCACGGCAGTTCC

Abbreviation: ARlncRNAs, anoikis-related lncRNAs; RT-qPCR, Reverse transcription quantitative-polymerase chain reaction.

Supplementary Table 2. The 148 ARlncRNAs connected with the over survival of the LUAD patients screened after univariate Cox analysis in training cohort (P<0.05).

Gene	HR	HR (95% CI)	P-value	Gene	HR	HR (95% CI)	P-value
MIR4435-2HG	1.68	(1.17,2.42)	0.005	AL445309.1	0.59	(0.39,0.91)	0.016
AC103681.2	0.29	(0.10,0.81)	0.018	AC092718.4	1.45	(1.15,1.85)	0.002
AC107308.1	1.30	(1.05,1.61)	0.016	Z97989.1	0.61	(0.38,0.98)	0.042
TSPOAP1-AS1	0.65	(0.44,0.98)	0.039	AC026356.1	1.34	(1.03,1.76)	0.030
AC039056.2	1.40	(1.05,1.88)	0.022	ABCA9-AS1	2.10	(1.14,3.87)	0.017
LINC01863	0.68	(0.48,0.96)	0.027	C2orf27A	1.50	(1.06,2.13)	0.023
CYP1B1-AS1	0.51	(0.27,0.96)	0.038	AC079313.2	2.46	(1.47,4.13)	0.001
AL109615.4	0.71	(0.54,0.94)	0.017	SMILR	1.60	(1.19,2.15)	0.002
AC087501.4	0.43	(0.25,0.75)	0.003	AC116407.1	0.80	(0.64,0.99)	0.045
AC092279.1	0.73	(0.56,0.96)	0.022	AC006116.9	0.21	(0.05,0.88)	0.033
AC022148.2	0.30	(0.10,0.90)	0.032	GMDS-DT	0.40	(0.20,0.80)	0.009
ZKSCAN7-AS1	0.59	(0.38,0.92)	0.021	LINC01116	1.28	(1.09,1.49)	0.002
AC009509.4	1.75	(1.17,2.63)	0.007	AC092574.1	0.65	(0.45,0.93)	0.020
CACTIN-AS1	2.48	(1.04,5.92)	0.041	AL606489.1	1.43	(1.16,1.77)	0.001
AC090948.1	0.72	(0.53,0.98)	0.034	DEPDC1-AS1	4.28	(1.89,9.66)	<0.001
AC090023.2	1.85	(1.24,2.75)	0.003	AL449423.1	1.67	(1.03,2.72)	0.037
LINC01138	1.42	(1.00,2.00)	0.047	AL691432.2	0.62	(0.47,0.84)	0.002
AC018529.1	0.58	(0.34,0.98)	0.042	ZNF571-AS1	0.63	(0.41,0.97)	0.035
AC011477.2	0.70	(0.54,0.92)	0.009	RASAL2-AS1	1.56	(1.01,2.40)	0.043
AC007552.2	0.77	(0.61,0.97)	0.024	AL157895.1	0.48	(0.25,0.94)	0.032
SH3BP5-AS1	0.75	(0.58,0.97)	0.027	AL358115.1	1.60	(1.03,2.49)	0.035
SNHG14	0.67	(0.45,0.99)	0.042	AC025171.2	0.74	(0.57,0.97)	0.029
APCDD1L-DT	1.53	(1.20,1.94)	0.001	AC112721.1	1.34	(1.01,1.77)	0.040
AC124242.1	0.51	(0.29,0.89)	0.019	DAAM2-AS1	0.40	(0.18,0.91)	0.029
AC027031.2	1.37	(1.09,1.72)	0.006	AC010615.2	0.79	(0.62,1.00)	0.047
CYTOR	1.62	(1.11,2.36)	0.013	TMEM30A-DT	0.27	(0.11,0.68)	0.006
ADAMTS9-AS2	0.47	(0.25,0.89)	0.020	AL137009.1	0.64	(0.43,0.94)	0.021

AC003991.1	0.57	(0.33,0.98)	0.041	AL359220.1	0.58	(0.36,0.93)	0.022
LINC00520	1.80	(1.20,2.69)	0.004	ZNF32-AS2	0.73	(0.54,0.99)	0.045
LINC00460	1.18	(1.02,1.37)	0.026	AC125807.2	1.33	(1.01,1.74)	0.041
AC107021.2	1.25	(1.00,1.57)	0.047	LINC02310	2.75	(1.83,4.14)	<0.001
AL008729.2	0.80	(0.66,0.97)	0.025	AC138965.1	1.63	(1.07,2.48)	0.023
AL031602.2	0.53	(0.36,0.78)	0.001	STXBP5-AS1	2.45	(1.11,5.42)	0.027
BZW1-AS1	2.43	(1.58,3.74)	<0.001	AC034223.2	1.53	(1.28,1.85)	<0.001
AC008937.3	0.46	(0.23,0.93)	0.030	AP005264.1	1.91	(1.21,3.03)	0.006
AC100810.3	1.68	(1.15,2.47)	0.007	AP005717.2	2.35	(1.01,5.48)	0.047
LINC02728	0.43	(0.21,0.90)	0.025	AC010343.3	1.54	(1.07,2.21)	0.019
AC092718.5	0.58	(0.38,0.91)	0.017	AC024075.1	0.76	(0.6,0.97)	0.025
AC021087.1	0.57	(0.38,0.84)	0.005	AL162632.3	2.96	(1.22,7.18)	0.016
AC084048.1	0.45	(0.21,0.97)	0.042	AC024075.3	0.72	(0.53,0.97)	0.031
AC004540.2	0.59	(0.37,0.94)	0.026	AC091057.1	1.35	(1.03,1.78)	0.030
AC021087.3	0.58	(0.37,0.93)	0.023	AC022210.1	1.53	(1.09,2.15)	0.013
AL137186.2	1.73	(1.04,2.86)	0.034	AC090409.1	1.88	(1.00,3.55)	0.050
GLIS2-AS1	0.62	(0.45,0.84)	0.002	ZNF790-AS1	0.62	(0.42,0.92)	0.017
LINC02848	0.06	(0.01,0.74)	0.028	AL356608.1	0.13	(0.03,0.56)	0.006
TMPO-AS1	1.38	(1.02,1.86)	0.034	LINC01117	1.88	(1.41,2.50)	<0.001
AL354953.1	1.31	(1.11,1.55)	0.002	AC008870.2	0.73	(0.53,0.99)	0.045
STEAP2-AS1	1.76	(1.13,2.73)	0.012	AC108136.1	1.97	(1.37,2.83)	<0.001
ZRANB2-AS2	0.17	(0.04,0.74)	0.018	AL353804.1	0.73	(0.56,0.96)	0.026
AC091435.2	0.61	(0.38,0.99)	0.047	AL139351.3	1.77	(1.23,2.55)	0.002
AC124045.1	0.67	(0.46,1.00)	0.048	ASB16-AS1	0.69	(0.47,0.99)	0.046
RMDN2-AS1	0.58	(0.35,0.96)	0.034	LINC01537	2.40	(1.60,3.60)	<0.001
AP000864.1	2.22	(1.11,4.43)	0.024	AC006058.3	1.57	(1.15,2.16)	0.005
AC026355.2	0.81	(0.67,0.98)	0.034	AC123595.2	0.61	(0.41,0.90)	0.014
AC092640.1	1.94	(1.12,3.36)	0.018	AC005865.2	1.40	(1.05,1.88)	0.024
AL138689.1	1.37	(1.06,1.77)	0.018	HAS2-AS1	5.20	(2.48,10.92)	<0.001
AC084781.1	2.79	(1.20,6.47)	0.017	AC092329.4	0.53	(0.33,0.87)	0.011
AL031778.1	0.64	(0.44,0.92)	0.017	AP000695.1	1.53	(1.23,1.91)	<0.001
AP000695.2	1.48	(1.16,1.88)	0.001	AL138921.2	0.66	(0.44,0.98)	0.039
AC135050.6	0.60	(0.44,0.82)	0.001	AL138789.1	1.53	(1.15,2.02)	0.003
AL109811.2	0.61	(0.44,0.84)	0.003	AC025419.1	1.51	(1.15,1.98)	0.003
LINC01711	1.40	(1.13,1.74)	0.002	AC024909.1	0.61	(0.38,0.98)	0.041
TRMT2B-AS1	0.56	(0.34,0.94)	0.028	AC068580.2	1.41	(1.00,1.98)	0.047
MANCR	1.68	(1.34,2.10)	<0.001	AL355075.2	0.76	(0.59,0.98)	0.035
AL139424.3	0.42	(0.18,0.95)	0.037	NAGPA-AS1	0.64	(0.42,0.98)	0.042
AC127024.5	0.69	(0.50,0.97)	0.030	HCG18	0.53	(0.34,0.85)	0.007
AL158068.2	1.95	(1.02,3.70)	0.042	AL391261.1	2.08	(1.33,3.26)	0.001
NIPBL-DT	0.69	(0.50,0.95)	0.023	MAP3K4-AS1	1.55	(1.09,2.21)	0.016
AC016735.1	1.37	(1.03,1.82)	0.030	AL359643.2	0.26	(0.08,0.91)	0.035
AC019205.1	0.56	(0.32,0.97)	0.038	AC114781.2	0.45	(0.24,0.84)	0.013
LINC01385	1.33	(1.03,1.72)	0.029	AC060780.1	0.70	(0.50,0.97)	0.033
AC034102.8	0.62	(0.41,0.92)	0.019	AL590729.1	0.57	(0.35,0.92)	0.022
DIRC3	2.27	(1.20,4.30)	0.012	SEPSECS-AS1	0.62	(0.39,0.99)	0.045
MIR31HG	1.54	(1.30,1.81)	<0.001	LINC02582	1.41	(1.11,1.78)	0.005

Abbreviation: ARlncRNAs, anoikis-related lncRNAs; CI, Confidence interval; HR, Hazard ratios; LUAD, Lung adenocarcinoma.

Supplementary Table 3. 11 ARlncRNAs connected with the over survival of the LUAD patients after multivariate Cox analysis in training cohort.

Gene	HR	HR (95% CI)	Coef	P-value
AL031602.2	0.49	(0.32,0.77)	-0.710450178205890	0.002
BZW1-AS1	1.54	(0.93,2.54)	0.432038348321673	0.092
AC021087.1	0.62	(0.41,0.94)	-0.476411291926875	0.025
GLIS2-AS1	0.64	(0.46,0.87)	-0.454008732783369	0.005
ABCA9-AS1	2.72	(1.31,5.63)	0.999706491359515	0.007
AL606489.1	1.30	(1.03,1.63)	0.259835252523394	0.025
ZNF571-AS1	0.71	(0.44,1.12)	-0.348748604018090	0.139
LINC02310	2.12	(1.24,3.62)	0.750892025449650	0.006
AL162632.3	3.73	(1.43,9.72)	1.315573571091090	0.007
LINC01117	1.40	(1.03,1.90)	0.338762422003907	0.029
HAS2-AS1	2.17	(0.93,5.05)	0.775846321855369	0.072

Abbreviations: ARlncRNAs, anoikis-related lncRNAs; CI, Confidence interval; Coef, Coefficient; HR, Hazard ratios; LUAD, Lung adenocarcinoma.
