# Supplementary material for: Prognostic value and immune landscapes of anoikis-associated lncRNAs in lung adenocarcinoma
Source: Aging (Albany NY). 2024 Feb 5;16(3):2273–98. doi: 10.18632/aging.205481 (PMC10911388; doi:10.18632/aging.205481)
Supplement: Supplementary Table 4 [file aging-16-205481-s003.doc]

Supplementary Table 4. The risk scores and risk groups of LUAD patients in the total cohort.

| **Patient ID** | **Risk score** | **Group** |  | **Patient ID** | **Risk score** | **Group** |
| --- | --- | --- | --- | --- | --- | --- |
| TCGA-62-A471 | 2.710452172 | High risk |  | TCGA-35-5375 | 1.519315303 | High risk |
| TCGA-L9-A5IP | 9.415268843 | High risk |  | TCGA-55-A4DF | 0.23908903 | Low risk |
| TCGA-44-7659 | 0.307491064 | Low risk |  | TCGA-MP-A4T4 | 0.958841184 | High risk |
| TCGA-62-A46P | 1.238407968 | High risk |  | TCGA-49-AARR | 0.329025802 | Low risk |
| TCGA-86-8073 | 0.378352924 | Low risk |  | TCGA-78-8662 | 3.914257274 | High risk |
| TCGA-69-7765 | 0.994711999 | High risk |  | TCGA-05-4430 | 0.821885053 | Low risk |
| TCGA-55-6984 | 2.751317419 | High risk |  | TCGA-L9-A50W | 0.465912674 | Low risk |
| TCGA-86-A4P7 | 0.309498648 | Low risk |  | TCGA-75-7025 | 0.561916806 | Low risk |
| TCGA-97-8177 | 1.156134476 | High risk |  | TCGA-05-4424 | 1.789303376 | High risk |
| TCGA-50-6595 | 1.498305804 | High risk |  | TCGA-38-4627 | 3.042507437 | High risk |
| TCGA-78-7633 | 1.025285736 | High risk |  | TCGA-44-5645 | 0.758866018 | Low risk |
| TCGA-97-A4LX | 0.662147541 | Low risk |  | TCGA-69-7764 | 0.980356945 | High risk |
| TCGA-53-7624 | 3.996552594 | High risk |  | TCGA-55-8620 | 0.777216163 | Low risk |
| TCGA-62-A46R | 1.107107905 | High risk |  | TCGA-55-A492 | 0.373494847 | Low risk |
| TCGA-78-7156 | 2.734433348 | High risk |  | TCGA-78-7220 | 5.787361098 | High risk |
| TCGA-50-5072 | 16.63455389 | High risk |  | TCGA-49-AARN | 0.815613899 | Low risk |
| TCGA-91-6831 | 1.641653336 | High risk |  | TCGA-50-8457 | 0.389290553 | Low risk |
| TCGA-91-A4BD | 0.615532671 | Low risk |  | TCGA-91-8496 | 0.517686963 | Low risk |
| TCGA-69-8253 | 0.171728664 | Low risk |  | TCGA-55-8092 | 5.074677732 | High risk |
| TCGA-86-A4D0 | 2.606547978 | High risk |  | TCGA-NJ-A4YQ | 1.518233424 | High risk |
| TCGA-55-8096 | 0.832622956 | Low risk |  | TCGA-69-8255 | 0.849999277 | Low risk |
| TCGA-44-2662 | 0.893515654 | Low risk |  | TCGA-78-8640 | 2.013536985 | High risk |
| TCGA-86-A4P8 | 0.359222062 | Low risk |  | TCGA-97-8174 | 0.561846672 | Low risk |
| TCGA-78-7158 | 0.486078377 | Low risk |  | TCGA-55-6987 | 0.915949763 | Low risk |
| TCGA-44-4112 | 1.021879021 | High risk |  | TCGA-49-6744 | 1.677887655 | High risk |
| TCGA-95-7948 | 0.275761462 | Low risk |  | TCGA-J2-8192 | 1.717402777 | High risk |
| TCGA-05-4432 | 1.219715268 | High risk |  | TCGA-44-7671 | 0.896740225 | Low risk |
| TCGA-78-7143 | 0.341672146 | Low risk |  | TCGA-49-AAR9 | 15.13516774 | High risk |
| TCGA-91-8499 | 0.652330196 | Low risk |  | TCGA-80-5611 | 0.242650561 | Low risk |
| TCGA-05-4434 | 10.43074712 | High risk |  | TCGA-71-6725 | 1.44518679 | High risk |
| TCGA-67-3773 | 0.348270867 | Low risk |  | TCGA-44-2656 | 1.683119797 | High risk |
| TCGA-05-5425 | 2.066432963 | High risk |  | TCGA-05-5715 | 2.622767691 | High risk |
| TCGA-97-A4M2 | 0.399482401 | Low risk |  | TCGA-64-5815 | 1.097062448 | High risk |
| TCGA-50-5941 | 1.141100661 | High risk |  | TCGA-55-7816 | 1.053414272 | High risk |
| TCGA-78-7539 | 0.124727492 | Low risk |  | TCGA-99-8033 | 2.31878274 | High risk |
| TCGA-05-5429 | 3.381792515 | High risk |  | TCGA-73-4668 | 1.123663367 | High risk |
| TCGA-93-A4JO | 0.661097028 | Low risk |  | TCGA-44-5644 | 0.467024882 | Low risk |
| TCGA-86-A4JF | 1.258415128 | High risk |  | TCGA-38-4625 | 1.219059171 | High risk |
| TCGA-55-7914 | 1.046314364 | High risk |  | TCGA-86-6851 | 1.147507292 | High risk |
| TCGA-MP-A4TC | 1.825719502 | High risk |  | TCGA-44-6779 | 3.892100994 | High risk |
| TCGA-64-1681 | 0.420614192 | Low risk |  | TCGA-78-7537 | 0.89118962 | Low risk |
| TCGA-05-4249 | 0.683199703 | Low risk |  | TCGA-67-3771 | 0.520330545 | Low risk |
| TCGA-91-A4BC | 1.669463046 | High risk |  | TCGA-55-8299 | 1.396701106 | High risk |
| TCGA-55-7570 | 0.531027114 | Low risk |  | TCGA-44-A47A | 0.840994712 | Low risk |
| TCGA-86-8672 | 1.644703468 | High risk |  | TCGA-05-4402 | 0.992668707 | High risk |
| TCGA-50-5942 | 0.385439686 | Low risk |  | TCGA-49-4490 | 1.489930655 | High risk |
| TCGA-50-6592 | 5.965559752 | High risk |  | TCGA-55-8090 | 1.144970896 | High risk |
| TCGA-62-8395 | 0.183501503 | Low risk |  | TCGA-99-AA5R | 0.496832301 | Low risk |
| TCGA-05-4410 | 0.403371912 | Low risk |  | TCGA-49-4486 | 0.391472482 | Low risk |
| TCGA-55-6975 | 3.412013529 | High risk |  | TCGA-91-6847 | 3.712625309 | High risk |
| TCGA-73-4662 | 0.183835862 | Low risk |  | TCGA-50-5049 | 1.272490692 | High risk |
| TCGA-49-6767 | 2.744051539 | High risk |  | TCGA-50-5066 | 0.727538145 | Low risk |
| TCGA-NJ-A4YG | 0.461336154 | Low risk |  | TCGA-55-7727 | 0.892786438 | Low risk |
| TCGA-NJ-A7XG | 0.722147479 | Low risk |  | TCGA-86-8674 | 0.155452839 | Low risk |
| TCGA-44-6774 | 0.885823885 | Low risk |  | TCGA-69-A59K | 0.920164628 | Low risk |
| TCGA-49-AAR4 | 1.510820116 | High risk |  | TCGA-44-2666 | 1.153653545 | High risk |
| TCGA-44-2668 | 0.638779785 | Low risk |  | TCGA-44-A47G | 0.783620257 | Low risk |
| TCGA-55-8091 | 0.702672754 | Low risk |  | TCGA-55-8507 | 0.426047819 | Low risk |
| TCGA-49-AARE | 1.540119265 | High risk |  | TCGA-05-4433 | 1.802148699 | High risk |
| TCGA-55-6985 | 0.796496436 | Low risk |  | TCGA-55-A4DG | 0.507338675 | Low risk |
| TCGA-05-4427 | 0.649383142 | Low risk |  | TCGA-49-4494 | 4.527774869 | High risk |
| TCGA-49-4487 | 1.250314216 | High risk |  | TCGA-44-6147 | 1.056379445 | High risk |
| TCGA-49-4507 | 6.404198804 | High risk |  | TCGA-49-AARO | 0.932256831 | Low risk |
| TCGA-55-8514 | 0.138130228 | Low risk |  | TCGA-91-6848 | 22.62085344 | High risk |
| TCGA-55-7576 | 1.503338962 | High risk |  | TCGA-50-8460 | 0.66430391 | Low risk |
| TCGA-55-6969 | 0.998194465 | High risk |  | TCGA-50-6673 | 1.228479621 | High risk |
| TCGA-69-7978 | 2.253802445 | High risk |  | TCGA-55-6642 | 1.454433943 | High risk |
| TCGA-NJ-A4YF | 0.216664288 | Low risk |  | TCGA-50-6591 | 1.992680383 | High risk |
| TCGA-91-6830 | 2.433641215 | High risk |  | TCGA-78-7166 | 1.019957259 | High risk |
| TCGA-78-7540 | 3.835751472 | High risk |  | TCGA-73-4676 | 0.881732656 | Low risk |
| TCGA-99-8028 | 0.702518202 | Low risk |  | TCGA-78-7148 | 3.07995466 | High risk |
| TCGA-64-1677 | 0.679438024 | Low risk |  | TCGA-93-7347 | 0.958143232 | High risk |
| TCGA-55-6980 | 1.045931031 | High risk |  | TCGA-44-7672 | 1.978271234 | High risk |
| TCGA-55-8615 | 1.146918126 | High risk |  | TCGA-55-7227 | 1.001545941 | High risk |
| TCGA-55-A494 | 1.030202344 | High risk |  | TCGA-MP-A4T6 | 0.154346387 | Low risk |
| TCGA-78-7147 | 0.480638506 | Low risk |  | TCGA-05-4403 | 1.430437665 | High risk |
| TCGA-55-7573 | 0.535494564 | Low risk |  | TCGA-55-8511 | 0.984568861 | High risk |
| TCGA-64-1679 | 0.369290284 | Low risk |  | TCGA-78-7167 | 3.933678568 | High risk |
| TCGA-64-1680 | 0.961626921 | High risk |  | TCGA-97-A4M0 | 0.444047596 | Low risk |
| TCGA-99-8025 | 0.814546389 | Low risk |  | TCGA-86-7714 | 0.885151007 | Low risk |
| TCGA-L9-A743 | 0.494519289 | Low risk |  | TCGA-86-8669 | 1.934811346 | High risk |
| TCGA-55-8094 | 0.490011236 | Low risk |  | TCGA-78-7145 | 1.996949566 | High risk |
| TCGA-44-3919 | 0.379930124 | Low risk |  | TCGA-86-8075 | 0.903134355 | Low risk |
| TCGA-80-5608 | 2.207391327 | High risk |  | TCGA-86-A456 | 0.24292327 | Low risk |
| TCGA-67-3772 | 1.391570436 | High risk |  | TCGA-MP-A4SW | 0.30987958 | Low risk |
| TCGA-35-4122 | 2.686209721 | High risk |  | TCGA-69-7973 | 7.368713963 | High risk |
| TCGA-55-6982 | 2.886664787 | High risk |  | TCGA-86-8671 | 0.755501143 | Low risk |
| TCGA-05-4396 | 5.954727067 | High risk |  | TCGA-35-4123 | 1.848422428 | High risk |
| TCGA-55-7815 | 0.577304254 | Low risk |  | TCGA-49-6761 | 0.912619853 | Low risk |
| TCGA-MN-A4N1 | 0.191862112 | Low risk |  | TCGA-44-A4SU | 0.759612685 | Low risk |
| TCGA-55-8301 | 1.835230493 | High risk |  | TCGA-86-8279 | 0.665585091 | Low risk |
| TCGA-62-A46O | 3.279836388 | High risk |  | TCGA-86-8358 | 1.197034918 | High risk |
| TCGA-97-8552 | 0.185455898 | Low risk |  | TCGA-78-7162 | 1.657837513 | High risk |
| TCGA-MP-A5C7 | 0.224896487 | Low risk |  | TCGA-44-6777 | 1.072287483 | High risk |
| TCGA-97-8175 | 0.619696489 | Low risk |  | TCGA-05-4422 | 1.115896168 | High risk |
| TCGA-91-8497 | 0.984917013 | High risk |  | TCGA-49-4506 | 11.83099461 | High risk |
| TCGA-38-4632 | 1.030367366 | High risk |  | TCGA-05-4417 | 0.885364798 | Low risk |
| TCGA-55-6972 | 1.117664129 | High risk |  | TCGA-75-5146 | 0.71147609 | Low risk |
| TCGA-55-7724 | 1.071251107 | High risk |  | TCGA-86-8074 | 1.243806138 | High risk |
| TCGA-69-7980 | 0.675321264 | Low risk |  | TCGA-97-7937 | 0.17060391 | Low risk |
| TCGA-73-7498 | 0.524039101 | Low risk |  | TCGA-97-A4M7 | 0.464971668 | Low risk |
| TCGA-86-8585 | 0.44546204 | Low risk |  | TCGA-05-4420 | 1.542065346 | High risk |
| TCGA-78-7542 | 5.805697983 | High risk |  | TCGA-78-7163 | 0.27649761 | Low risk |
| TCGA-38-4630 | 0.547053658 | Low risk |  | TCGA-86-7701 | 2.088965334 | High risk |
| TCGA-55-8085 | 0.949696812 | Low risk |  | TCGA-67-3770 | 0.917843415 | Low risk |
| TCGA-55-7284 | 1.182107474 | High risk |  | TCGA-38-4629 | 3.10908434 | High risk |
| TCGA-62-A472 | 1.53272566 | High risk |  | TCGA-MP-A4TA | 5.992050762 | High risk |
| TCGA-MP-A4TI | 3.436692878 | High risk |  | TCGA-50-5931 | 0.670459128 | Low risk |
| TCGA-44-8120 | 0.739713936 | Low risk |  | TCGA-55-7994 | 1.503576737 | High risk |
| TCGA-O1-A52J | 1.447446844 | High risk |  | TCGA-55-7725 | 0.177138552 | Low risk |
| TCGA-MP-A4TK | 2.69459258 | High risk |  | TCGA-MP-A4SY | 2.503577572 | High risk |
| TCGA-73-4666 | 1.555877132 | High risk |  | TCGA-78-7154 | 2.762392315 | High risk |
| TCGA-55-7281 | 0.445093819 | Low risk |  | TCGA-73-4659 | 0.910718063 | Low risk |
| TCGA-86-8668 | 0.374287692 | Low risk |  | TCGA-50-5068 | 0.655158459 | Low risk |
| TCGA-50-5939 | 13.56157119 | High risk |  | TCGA-05-4250 | 1.800789562 | High risk |
| TCGA-49-AAR3 | 1.57323185 | High risk |  | TCGA-44-6776 | 1.335154997 | High risk |
| TCGA-05-4395 | 994.9136941 | High risk |  | TCGA-05-4418 | 3.92747995 | High risk |
| TCGA-49-4514 | 1.482500569 | High risk |  | TCGA-55-7995 | 0.896567366 | Low risk |
| TCGA-62-A46S | 0.38942436 | Low risk |  | TCGA-55-6979 | 1.041624477 | High risk |
| TCGA-55-A490 | 1.327999196 | High risk |  | TCGA-55-8204 | 0.924349126 | Low risk |
| TCGA-55-8097 | 0.219788912 | Low risk |  | TCGA-62-8398 | 8.62856684 | High risk |
| TCGA-55-8208 | 0.969859986 | High risk |  | TCGA-67-4679 | 0.210817737 | Low risk |
| TCGA-55-6968 | 1.470410731 | High risk |  | TCGA-05-4390 | 3.252457308 | High risk |
| TCGA-44-6145 | 1.374495537 | High risk |  | TCGA-67-6217 | 0.144183983 | Low risk |
| TCGA-44-7667 | 1.901480871 | High risk |  | TCGA-55-5899 | 0.915034815 | Low risk |
| TCGA-97-7553 | 0.514817985 | Low risk |  | TCGA-MN-A4N4 | 0.31391781 | Low risk |
| TCGA-55-8089 | 7.975096571 | High risk |  | TCGA-L4-A4E6 | 0.949683813 | Low risk |
| TCGA-49-4501 | 0.848430201 | Low risk |  | TCGA-78-7536 | 1.285077767 | High risk |
| TCGA-38-4628 | 1.978247088 | High risk |  | TCGA-05-4389 | 2.76198784 | High risk |
| TCGA-55-A48Y | 1.308275632 | High risk |  | TCGA-97-7547 | 0.877634535 | Low risk |
| TCGA-MP-A4T9 | 0.471567988 | Low risk |  | TCGA-62-8394 | 0.809533688 | Low risk |
| TCGA-91-6835 | 0.822165067 | Low risk |  | TCGA-38-7271 | 1.558349221 | High risk |
| TCGA-50-6590 | 2.913547771 | High risk |  | TCGA-49-AARQ | 0.60956373 | Low risk |
| TCGA-50-5051 | 1.171500203 | High risk |  | TCGA-64-1678 | 1.792640008 | High risk |
| TCGA-97-A4M3 | 0.183073816 | Low risk |  | TCGA-44-A479 | 0.490089348 | Low risk |
| TCGA-MP-A4SV | 0.976148377 | High risk |  | TCGA-97-7938 | 0.19369866 | Low risk |
| TCGA-35-3615 | 0.144624144 | Low risk |  | TCGA-69-7974 | 0.909591369 | Low risk |
| TCGA-97-A4M6 | 0.980900453 | High risk |  | TCGA-44-3917 | 0.784180833 | Low risk |
| TCGA-86-7955 | 1.64381497 | High risk |  | TCGA-05-4415 | 6.550874695 | High risk |
| TCGA-55-A491 | 1.126291057 | High risk |  | TCGA-78-7150 | 5.478676462 | High risk |
| TCGA-86-8056 | 0.136471062 | Low risk |  | TCGA-69-7763 | 0.407101001 | Low risk |
| TCGA-78-8655 | 1.03568078 | High risk |  | TCGA-95-7947 | 1.347924137 | High risk |
| TCGA-L9-A444 | 0.232092478 | Low risk |  | TCGA-86-7711 | 0.78829928 | Low risk |
| TCGA-86-8055 | 9.020951266 | High risk |  | TCGA-55-8621 | 0.753887844 | Low risk |
| TCGA-49-6745 | 1.112750127 | High risk |  | TCGA-L9-A8F4 | 0.262079705 | Low risk |
| TCGA-05-4405 | 0.48344905 | Low risk |  | TCGA-86-7953 | 1.493241415 | High risk |
| TCGA-MP-A4TH | 0.383642948 | Low risk |  | TCGA-95-7039 | 0.60934787 | Low risk |
| TCGA-55-1594 | 0.437557549 | Low risk |  | TCGA-55-8206 | 0.498243561 | Low risk |
| TCGA-44-7669 | 0.894678879 | Low risk |  | TCGA-44-7661 | 2.91144144 | High risk |
| TCGA-55-7913 | 3.13204009 | High risk |  | TCGA-J2-A4AD | 0.676688451 | Low risk |
| TCGA-95-7944 | 1.974378175 | High risk |  | TCGA-75-6206 | 1.187831889 | High risk |
| TCGA-50-5933 | 1.698477295 | High risk |  | TCGA-55-8616 | 1.612205096 | High risk |
| TCGA-91-7771 | 0.571838154 | Low risk |  | TCGA-97-7552 | 1.345420728 | High risk |
| TCGA-73-4675 | 1.954743678 | High risk |  | TCGA-44-2657 | 0.396977591 | Low risk |
| TCGA-55-6981 | 1.958463633 | High risk |  | TCGA-91-6840 | 2.304860908 | High risk |
| TCGA-75-5147 | 1.378845746 | High risk |  | TCGA-55-7907 | 1.578479808 | High risk |
| TCGA-L9-A7SV | 0.215349147 | Low risk |  | TCGA-49-4510 | 0.254314311 | Low risk |
| TCGA-44-3918 | 0.680316455 | Low risk |  | TCGA-55-7726 | 8.396340529 | High risk |
| TCGA-38-6178 | 1.415150725 | High risk |  | TCGA-L9-A443 | 0.259649193 | Low risk |
| TCGA-78-7535 | 0.558268917 | Low risk |  | TCGA-53-7813 | 0.316890339 | Low risk |
| TCGA-55-6986 | 0.700802153 | Low risk |  | TCGA-55-6970 | 2.064363929 | High risk |
| TCGA-91-6849 | 0.101140511 | Low risk |  | TCGA-99-7458 | 0.334336997 | Low risk |
| TCGA-86-8280 | 0.849394521 | Low risk |  | TCGA-86-6562 | 1.607214667 | High risk |
| TCGA-91-6828 | 2.311284945 | High risk |  | TCGA-05-4398 | 0.945638912 | Low risk |
| TCGA-50-5932 | 2.206186859 | High risk |  | TCGA-78-7161 | 0.809067248 | Low risk |
| TCGA-05-5423 | 0.388288061 | Low risk |  | TCGA-55-8087 | 0.345001147 | Low risk |
| TCGA-05-5420 | 1.500456758 | High risk |  | TCGA-67-6216 | 0.346342876 | Low risk |
| TCGA-62-A46Y | 0.22275616 | Low risk |  | TCGA-64-1676 | 0.421099717 | Low risk |
| TCGA-55-7910 | 0.362997568 | Low risk |  | TCGA-78-7160 | 0.540932174 | Low risk |
| TCGA-MN-A4N5 | 2.932427588 | High risk |  | TCGA-55-1595 | 0.313750196 | Low risk |
| TCGA-95-A4VK | 0.89767564 | Low risk |  | TCGA-62-8402 | 0.48794345 | Low risk |
| TCGA-95-8494 | 1.92837254 | High risk |  | TCGA-78-8648 | 1.114799689 | High risk |
| TCGA-55-1592 | 1.209510972 | High risk |  | TCGA-55-A57B | 0.356143008 | Low risk |
| TCGA-05-4397 | 3.268027924 | High risk |  | TCGA-55-7903 | 0.669347433 | Low risk |
| TCGA-86-8359 | 4.021785959 | High risk |  | TCGA-55-A493 | 2.123236225 | High risk |
| TCGA-75-6212 | 0.660259267 | Low risk |  | TCGA-55-A48X | 1.534205121 | High risk |
| TCGA-J2-A4AG | 0.235518181 | Low risk |  | TCGA-05-4384 | 1.374295049 | High risk |
| TCGA-78-7152 | 0.79309396 | Low risk |  | TCGA-97-A4M1 | 0.502217305 | Low risk |
| TCGA-93-7348 | 0.374589874 | Low risk |  | TCGA-95-7567 | 1.219966615 | High risk |
| TCGA-64-5775 | 76.21792871 | High risk |  | TCGA-55-8302 | 1.996627782 | High risk |
| TCGA-78-7149 | 0.283820127 | Low risk |  | TCGA-75-7027 | 7.944329484 | High risk |
| TCGA-44-2661 | 0.579053365 | Low risk |  | TCGA-49-4505 | 0.863057757 | Low risk |
| TCGA-73-4658 | 1.551602569 | High risk |  | TCGA-44-6775 | 1.440885347 | High risk |
| TCGA-44-6778 | 0.337583937 | Low risk |  | TCGA-44-2665 | 1.951562817 | High risk |
| TCGA-55-8205 | 2.366378196 | High risk |  | TCGA-55-A48Z | 0.805466016 | Low risk |
| TCGA-55-8203 | 0.831498944 | Low risk |  | TCGA-44-3396 | 0.819910928 | Low risk |
| TCGA-78-8660 | 2.254423613 | High risk |  | TCGA-86-8281 | 0.126090248 | Low risk |
| TCGA-44-A47B | 0.43096967 | Low risk |  | TCGA-62-8397 | 0.795276213 | Low risk |
| TCGA-64-5778 | 0.174933194 | Low risk |  | TCGA-78-7153 | 0.712453863 | Low risk |
| TCGA-86-7954 | 0.664401404 | Low risk |  | TCGA-MP-A4T8 | 1.390369768 | High risk |
| TCGA-55-6543 | 0.554384847 | Low risk |  | TCGA-44-7670 | 0.656407907 | Low risk |
| TCGA-62-8399 | 0.043878507 | Low risk |  | TCGA-55-8512 | 0.237657215 | Low risk |
| TCGA-49-AAR0 | 0.473881901 | Low risk |  | TCGA-50-5946 | 0.702660052 | Low risk |
| TCGA-50-6593 | 1.103380613 | High risk |  | TCGA-38-4631 | 6.345181 | High risk |
| TCGA-67-3774 | 0.301698721 | Low risk |  | TCGA-73-7499 | 2.350681766 | High risk |
| TCGA-49-AAQV | 1.187730881 | High risk |  | TCGA-55-7283 | 1.098291476 | High risk |
| TCGA-71-8520 | 0.923259758 | Low risk |  | TCGA-38-4626 | 0.474197257 | Low risk |
| TCGA-55-8614 | 1.175882509 | High risk |  | TCGA-95-7562 | 2.36645911 | High risk |
| TCGA-55-7574 | 1.776629892 | High risk |  | TCGA-69-7760 | 2.201556148 | High risk |
| TCGA-44-7660 | 1.182639707 | High risk |  | TCGA-50-5935 | 0.645961745 | Low risk |
| TCGA-MP-A4T7 | 0.877841143 | Low risk |  | TCGA-95-7043 | 0.978474436 | High risk |
| TCGA-78-7155 | 0.515197334 | Low risk |  | TCGA-55-6971 | 2.589350728 | High risk |
| TCGA-MP-A4TE | 6.444112785 | High risk |  | TCGA-95-A4VN | 1.293404107 | High risk |
| TCGA-93-8067 | 1.805206251 | High risk |  | TCGA-J2-8194 | 0.572865337 | Low risk |
| TCGA-55-8510 | 0.734956836 | Low risk |  | TCGA-44-5643 | 2.14141348 | High risk |
| TCGA-86-7713 | 0.435158891 | Low risk |  | TCGA-44-A4SS | 4.451061701 | High risk |
| TCGA-NJ-A55R | 0.67261722 | Low risk |  | TCGA-97-8171 | 2.254055941 | High risk |
| TCGA-50-5044 | 10.01793318 | High risk |  | TCGA-93-A4JP | 0.631962921 | Low risk |
| TCGA-64-5774 | 0.876753721 | Low risk |  | TCGA-50-5930 | 1.846184199 | High risk |
| TCGA-55-6712 | 19.22676705 | High risk |  | TCGA-64-5781 | 2.914780694 | High risk |
| TCGA-44-2659 | 0.498939202 | Low risk |  | TCGA-69-8254 | 1.014347499 | High risk |
| TCGA-4B-A93V | 1.308676401 | High risk |  | TCGA-50-7109 | 0.836497594 | Low risk |
| TCGA-86-8076 | 0.586133608 | Low risk |  | TCGA-05-5428 | 1.448466191 | High risk |
| TCGA-83-5908 | 0.656131622 | Low risk |  | TCGA-NJ-A4YP | 1.178569109 | High risk |
| TCGA-L4-A4E5 | 1.205835468 | High risk |  | TCGA-44-8117 | 0.477350801 | Low risk |
| TCGA-S2-AA1A | 1.061890322 | High risk |  | TCGA-05-4425 | 1.326747651 | High risk |
| TCGA-55-6978 | 0.792443453 | Low risk |  | TCGA-55-1596 | 1.39703013 | High risk |
| TCGA-78-7146 | 35.78430229 | High risk |  | TCGA-91-6836 | 1.232198259 | High risk |
| TCGA-MP-A4TD | 1.855919857 | High risk |  | TCGA-99-8032 | 0.905087177 | Low risk |
| TCGA-78-7159 | 1.218999738 | High risk |  | TCGA-44-7662 | 0.884370889 | Low risk |
| TCGA-49-4512 | 0.616826166 | Low risk |  | TCGA-44-2655 | 0.686656917 | Low risk |
| TCGA-50-5045 | 1.111930207 | High risk |  | TCGA-MP-A4TF | 80.68835495 | High risk |
| TCGA-44-8119 | 2.362104642 | High risk |  | TCGA-67-6215 | 0.580252731 | Low risk |
| TCGA-49-4488 | 0.690866754 | Low risk |  | TCGA-55-7911 | 0.738546967 | Low risk |
| TCGA-49-6743 | 1.276743227 | High risk |  | TCGA-75-6214 | 5.740113698 | High risk |
| TCGA-86-8673 | 0.667663974 | Low risk |  | TCGA-73-4677 | 0.844138399 | Low risk |
| TCGA-05-4244 | 0.577741606 | Low risk |  | TCGA-97-7554 | 0.91136609 | Low risk |
| TCGA-73-4670 | 3.533691401 | High risk |  | TCGA-05-4426 | 0.834893226 | Low risk |
| TCGA-55-8506 | 0.657200741 | Low risk |  | TCGA-05-4382 | 0.508321347 | Low risk |
| TCGA-50-6597 | 0.366250927 | Low risk |  | TCGA-J2-A4AE | 0.184731142 | Low risk |
| TCGA-44-3398 | 0.941700642 | Low risk |  | TCGA-50-6594 | 3.210185698 | High risk |
| TCGA-73-A9RS | 0.874342604 | Low risk |  | TCGA-55-8505 | 2.247712654 | High risk |
| TCGA-53-7626 | 0.427309389 | Low risk |  | TCGA-75-5125 | 0.511260552 | Low risk |
| TCGA-97-A4M5 | 0.343890814 | Low risk |  | TCGA-62-A46V | 0.427911421 | Low risk |
| TCGA-62-A46U | 0.32444127 | Low risk |  | TCGA-91-6829 | 1.223892675 | High risk |
| TCGA-50-5055 | 0.859576652 | Low risk |  |  |  |  |
