# Supplementary material for: Prognostic value and immune landscapes of anoikis-associated lncRNAs in lung adenocarcinoma
Source: Aging (Albany NY). 2024 Feb 5;16(3):2273–98. doi: 10.18632/aging.205481 (PMC10911388; doi:10.18632/aging.205481)
Supplement: Supplementary Table 5 [file aging-16-205481-s004.doc]

Supplementary Table 5. The risk scores and risk groups of LUAD patients in the training cohort.

| **Patient ID** | **Risk score** | **Group** |  | **Patient ID** | **Risk score** | **Group** |
| --- | --- | --- | --- | --- | --- | --- |
| TCGA-62-A471 | 2.710452172 | High risk |  | TCGA-62-A46S | 0.38942436 | Low risk |
| TCGA-L9-A5IP | 9.415268843 | High risk |  | TCGA-55-A490 | 1.327999196 | High risk |
| TCGA-44-7659 | 0.307491064 | Low risk |  | TCGA-55-8097 | 0.219788912 | Low risk |
| TCGA-62-A46P | 1.238407968 | High risk |  | TCGA-55-8208 | 0.969859986 | High risk |
| TCGA-86-8073 | 0.378352924 | Low risk |  | TCGA-55-6968 | 1.470410731 | High risk |
| TCGA-69-7765 | 0.994711999 | High risk |  | TCGA-44-6145 | 1.374495537 | High risk |
| TCGA-55-6984 | 2.751317419 | High risk |  | TCGA-44-7667 | 1.901480871 | High risk |
| TCGA-86-A4P7 | 0.309498648 | Low risk |  | TCGA-97-7553 | 0.514817985 | Low risk |
| TCGA-97-8177 | 1.156134476 | High risk |  | TCGA-55-8089 | 7.975096571 | High risk |
| TCGA-50-6595 | 1.498305804 | High risk |  | TCGA-49-4501 | 0.848430201 | Low risk |
| TCGA-78-7633 | 1.025285736 | High risk |  | TCGA-38-4628 | 1.978247088 | High risk |
| TCGA-97-A4LX | 0.662147541 | Low risk |  | TCGA-55-A48Y | 1.308275632 | High risk |
| TCGA-53-7624 | 3.996552594 | High risk |  | TCGA-MP-A4T9 | 0.471567988 | Low risk |
| TCGA-62-A46R | 1.107107905 | High risk |  | TCGA-91-6835 | 0.822165067 | Low risk |
| TCGA-78-7156 | 2.734433348 | High risk |  | TCGA-50-6590 | 2.913547771 | High risk |
| TCGA-50-5072 | 16.63455389 | High risk |  | TCGA-50-5051 | 1.171500203 | High risk |
| TCGA-91-6831 | 1.641653336 | High risk |  | TCGA-97-A4M3 | 0.183073816 | Low risk |
| TCGA-91-A4BD | 0.615532671 | Low risk |  | TCGA-MP-A4SV | 0.976148377 | High risk |
| TCGA-69-8253 | 0.171728664 | Low risk |  | TCGA-35-3615 | 0.144624144 | Low risk |
| TCGA-86-A4D0 | 2.606547978 | High risk |  | TCGA-97-A4M6 | 0.980900453 | High risk |
| TCGA-55-8096 | 0.832622956 | Low risk |  | TCGA-86-7955 | 1.64381497 | High risk |
| TCGA-44-2662 | 0.893515654 | Low risk |  | TCGA-55-A491 | 1.126291057 | High risk |
| TCGA-86-A4P8 | 0.359222062 | Low risk |  | TCGA-86-8056 | 0.136471062 | Low risk |
| TCGA-78-7158 | 0.486078377 | Low risk |  | TCGA-78-8655 | 1.03568078 | High risk |
| TCGA-44-4112 | 1.021879021 | High risk |  | TCGA-L9-A444 | 0.232092478 | Low risk |
| TCGA-95-7948 | 0.275761462 | Low risk |  | TCGA-86-8055 | 9.020951266 | High risk |
| TCGA-05-4432 | 1.219715268 | High risk |  | TCGA-49-6745 | 1.112750127 | High risk |
| TCGA-78-7143 | 0.341672146 | Low risk |  | TCGA-05-4405 | 0.48344905 | Low risk |
| TCGA-91-8499 | 0.652330196 | Low risk |  | TCGA-MP-A4TH | 0.383642948 | Low risk |
| TCGA-05-4434 | 10.43074712 | High risk |  | TCGA-55-1594 | 0.437557549 | Low risk |
| TCGA-67-3773 | 0.348270867 | Low risk |  | TCGA-44-7669 | 0.894678879 | Low risk |
| TCGA-05-5425 | 2.066432963 | High risk |  | TCGA-55-7913 | 3.13204009 | High risk |
| TCGA-97-A4M2 | 0.399482401 | Low risk |  | TCGA-95-7944 | 1.974378175 | High risk |
| TCGA-50-5941 | 1.141100661 | High risk |  | TCGA-50-5933 | 1.698477295 | High risk |
| TCGA-78-7539 | 0.124727492 | Low risk |  | TCGA-91-7771 | 0.571838154 | Low risk |
| TCGA-05-5429 | 3.381792515 | High risk |  | TCGA-73-4675 | 1.954743678 | High risk |
| TCGA-93-A4JO | 0.661097028 | Low risk |  | TCGA-55-6981 | 1.958463633 | High risk |
| TCGA-86-A4JF | 1.258415128 | High risk |  | TCGA-75-5147 | 1.378845746 | High risk |
| TCGA-55-7914 | 1.046314364 | High risk |  | TCGA-L9-A7SV | 0.215349147 | Low risk |
| TCGA-MP-A4TC | 1.825719502 | High risk |  | TCGA-44-3918 | 0.680316455 | Low risk |
| TCGA-64-1681 | 0.420614192 | Low risk |  | TCGA-38-6178 | 1.415150725 | High risk |
| TCGA-05-4249 | 0.683199703 | Low risk |  | TCGA-78-7535 | 0.558268917 | Low risk |
| TCGA-91-A4BC | 1.669463046 | High risk |  | TCGA-55-6986 | 0.700802153 | Low risk |
| TCGA-55-7570 | 0.531027114 | Low risk |  | TCGA-91-6849 | 0.101140511 | Low risk |
| TCGA-86-8672 | 1.644703468 | High risk |  | TCGA-86-8280 | 0.849394521 | Low risk |
| TCGA-50-5942 | 0.385439686 | Low risk |  | TCGA-91-6828 | 2.311284945 | High risk |
| TCGA-50-6592 | 5.965559752 | High risk |  | TCGA-50-5932 | 2.206186859 | High risk |
| TCGA-62-8395 | 0.183501503 | Low risk |  | TCGA-05-5423 | 0.388288061 | Low risk |
| TCGA-05-4410 | 0.403371912 | Low risk |  | TCGA-05-5420 | 1.500456758 | High risk |
| TCGA-55-6975 | 3.412013529 | High risk |  | TCGA-62-A46Y | 0.22275616 | Low risk |
| TCGA-73-4662 | 0.183835862 | Low risk |  | TCGA-55-7910 | 0.362997568 | Low risk |
| TCGA-49-6767 | 2.744051539 | High risk |  | TCGA-MN-A4N5 | 2.932427588 | High risk |
| TCGA-NJ-A4YG | 0.461336154 | Low risk |  | TCGA-95-A4VK | 0.89767564 | Low risk |
| TCGA-NJ-A7XG | 0.722147479 | Low risk |  | TCGA-95-8494 | 1.92837254 | High risk |
| TCGA-44-6774 | 0.885823885 | Low risk |  | TCGA-55-1592 | 1.209510972 | High risk |
| TCGA-49-AAR4 | 1.510820116 | High risk |  | TCGA-05-4397 | 3.268027924 | High risk |
| TCGA-44-2668 | 0.638779785 | Low risk |  | TCGA-86-8359 | 4.021785959 | High risk |
| TCGA-55-8091 | 0.702672754 | Low risk |  | TCGA-75-6212 | 0.660259267 | Low risk |
| TCGA-49-AARE | 1.540119265 | High risk |  | TCGA-J2-A4AG | 0.235518181 | Low risk |
| TCGA-55-6985 | 0.796496436 | Low risk |  | TCGA-78-7152 | 0.79309396 | Low risk |
| TCGA-05-4427 | 0.649383142 | Low risk |  | TCGA-93-7348 | 0.374589874 | Low risk |
| TCGA-49-4487 | 1.250314216 | High risk |  | TCGA-64-5775 | 76.21792871 | High risk |
| TCGA-49-4507 | 6.404198804 | High risk |  | TCGA-78-7149 | 0.283820127 | Low risk |
| TCGA-55-8514 | 0.138130228 | Low risk |  | TCGA-44-2661 | 0.579053365 | Low risk |
| TCGA-55-7576 | 1.503338962 | High risk |  | TCGA-73-4658 | 1.551602569 | High risk |
| TCGA-55-6969 | 0.998194465 | High risk |  | TCGA-44-6778 | 0.337583937 | Low risk |
| TCGA-69-7978 | 2.253802445 | High risk |  | TCGA-55-8205 | 2.366378196 | High risk |
| TCGA-NJ-A4YF | 0.216664288 | Low risk |  | TCGA-55-8203 | 0.831498944 | Low risk |
| TCGA-91-6830 | 2.433641215 | High risk |  | TCGA-78-8660 | 2.254423613 | High risk |
| TCGA-78-7540 | 3.835751472 | High risk |  | TCGA-44-A47B | 0.43096967 | Low risk |
| TCGA-99-8028 | 0.702518202 | Low risk |  | TCGA-64-5778 | 0.174933194 | Low risk |
| TCGA-64-1677 | 0.679438024 | Low risk |  | TCGA-86-7954 | 0.664401404 | Low risk |
| TCGA-55-6980 | 1.045931031 | High risk |  | TCGA-55-6543 | 0.554384847 | Low risk |
| TCGA-55-8615 | 1.146918126 | High risk |  | TCGA-62-8399 | 0.043878507 | Low risk |
| TCGA-55-A494 | 1.030202344 | High risk |  | TCGA-49-AAR0 | 0.473881901 | Low risk |
| TCGA-78-7147 | 0.480638506 | Low risk |  | TCGA-50-6593 | 1.103380613 | High risk |
| TCGA-55-7573 | 0.535494564 | Low risk |  | TCGA-67-3774 | 0.301698721 | Low risk |
| TCGA-64-1679 | 0.369290284 | Low risk |  | TCGA-49-AAQV | 1.187730881 | High risk |
| TCGA-64-1680 | 0.961626921 | High risk |  | TCGA-71-8520 | 0.923259758 | Low risk |
| TCGA-99-8025 | 0.814546389 | Low risk |  | TCGA-55-8614 | 1.175882509 | High risk |
| TCGA-L9-A743 | 0.494519289 | Low risk |  | TCGA-55-7574 | 1.776629892 | High risk |
| TCGA-55-8094 | 0.490011236 | Low risk |  | TCGA-44-7660 | 1.182639707 | High risk |
| TCGA-44-3919 | 0.379930124 | Low risk |  | TCGA-MP-A4T7 | 0.877841143 | Low risk |
| TCGA-80-5608 | 2.207391327 | High risk |  | TCGA-78-7155 | 0.515197334 | Low risk |
| TCGA-67-3772 | 1.391570436 | High risk |  | TCGA-MP-A4TE | 6.444112785 | High risk |
| TCGA-35-4122 | 2.686209721 | High risk |  | TCGA-93-8067 | 1.805206251 | High risk |
| TCGA-55-6982 | 2.886664787 | High risk |  | TCGA-55-8510 | 0.734956836 | Low risk |
| TCGA-05-4396 | 5.954727067 | High risk |  | TCGA-86-7713 | 0.435158891 | Low risk |
| TCGA-55-7815 | 0.577304254 | Low risk |  | TCGA-NJ-A55R | 0.67261722 | Low risk |
| TCGA-MN-A4N1 | 0.191862112 | Low risk |  | TCGA-50-5044 | 10.01793318 | High risk |
| TCGA-55-8301 | 1.835230493 | High risk |  | TCGA-64-5774 | 0.876753721 | Low risk |
| TCGA-62-A46O | 3.279836388 | High risk |  | TCGA-55-6712 | 19.22676705 | High risk |
| TCGA-97-8552 | 0.185455898 | Low risk |  | TCGA-44-2659 | 0.498939202 | Low risk |
| TCGA-MP-A5C7 | 0.224896487 | Low risk |  | TCGA-4B-A93V | 1.308676401 | High risk |
| TCGA-97-8175 | 0.619696489 | Low risk |  | TCGA-86-8076 | 0.586133608 | Low risk |
| TCGA-91-8497 | 0.984917013 | High risk |  | TCGA-83-5908 | 0.656131622 | Low risk |
| TCGA-38-4632 | 1.030367366 | High risk |  | TCGA-L4-A4E5 | 1.205835468 | High risk |
| TCGA-55-6972 | 1.117664129 | High risk |  | TCGA-S2-AA1A | 1.061890322 | High risk |
| TCGA-55-7724 | 1.071251107 | High risk |  | TCGA-55-6978 | 0.792443453 | Low risk |
| TCGA-69-7980 | 0.675321264 | Low risk |  | TCGA-78-7146 | 35.78430229 | High risk |
| TCGA-73-7498 | 0.524039101 | Low risk |  | TCGA-MP-A4TD | 1.855919857 | High risk |
| TCGA-86-8585 | 0.44546204 | Low risk |  | TCGA-78-7159 | 1.218999738 | High risk |
| TCGA-78-7542 | 5.805697983 | High risk |  | TCGA-49-4512 | 0.616826166 | Low risk |
| TCGA-38-4630 | 0.547053658 | Low risk |  | TCGA-50-5045 | 1.111930207 | High risk |
| TCGA-55-8085 | 0.949696812 | Low risk |  | TCGA-44-8119 | 2.362104642 | High risk |
| TCGA-55-7284 | 1.182107474 | High risk |  | TCGA-49-4488 | 0.690866754 | Low risk |
| TCGA-62-A472 | 1.53272566 | High risk |  | TCGA-49-6743 | 1.276743227 | High risk |
| TCGA-MP-A4TI | 3.436692878 | High risk |  | TCGA-86-8673 | 0.667663974 | Low risk |
| TCGA-44-8120 | 0.739713936 | Low risk |  | TCGA-05-4244 | 0.577741606 | Low risk |
| TCGA-O1-A52J | 1.447446844 | High risk |  | TCGA-73-4670 | 3.533691401 | High risk |
| TCGA-MP-A4TK | 2.69459258 | High risk |  | TCGA-55-8506 | 0.657200741 | Low risk |
| TCGA-73-4666 | 1.555877132 | High risk |  | TCGA-50-6597 | 0.366250927 | Low risk |
| TCGA-55-7281 | 0.445093819 | Low risk |  | TCGA-44-3398 | 0.941700642 | Low risk |
| TCGA-86-8668 | 0.374287692 | Low risk |  | TCGA-73-A9RS | 0.874342604 | Low risk |
| TCGA-50-5939 | 13.56157119 | High risk |  | TCGA-53-7626 | 0.427309389 | Low risk |
| TCGA-49-AAR3 | 1.57323185 | High risk |  | TCGA-97-A4M5 | 0.343890814 | Low risk |
| TCGA-05-4395 | 994.9136941 | High risk |  | TCGA-62-A46U | 0.32444127 | Low risk |
| TCGA-49-4514 | 1.482500569 | High risk |  | TCGA-50-5055 | 0.859576652 | Low risk |
