# Supplementary material for: Prognostic value and immune landscapes of anoikis-associated lncRNAs in lung adenocarcinoma
Source: Aging (Albany NY). 2024 Feb 5;16(3):2273–98. doi: 10.18632/aging.205481 (PMC10911388; doi:10.18632/aging.205481)
Supplement: Supplementary Table 6 [file aging-16-205481-s005.doc]

Supplementary Table 6. The risk scores and risk groups of LUAD patients in the test cohort.

| **Patient ID** | **Risk score** | **Group** |  | **Patient ID** | **Risk score** | **Group** |
| --- | --- | --- | --- | --- | --- | --- |
| TCGA-35-5375 | 1.519315303 | High risk |  | TCGA-55-6979 | 1.041624477 | High risk |
| TCGA-55-A4DF | 0.23908903 | Low risk |  | TCGA-55-8204 | 0.924349126 | Low risk |
| TCGA-MP-A4T4 | 0.958841184 | High risk |  | TCGA-62-8398 | 8.62856684 | High risk |
| TCGA-49-AARR | 0.329025802 | Low risk |  | TCGA-67-4679 | 0.210817737 | Low risk |
| TCGA-78-8662 | 3.914257274 | High risk |  | TCGA-05-4390 | 3.252457308 | High risk |
| TCGA-05-4430 | 0.821885053 | Low risk |  | TCGA-67-6217 | 0.144183983 | Low risk |
| TCGA-L9-A50W | 0.465912674 | Low risk |  | TCGA-55-5899 | 0.915034815 | Low risk |
| TCGA-75-7025 | 0.561916806 | Low risk |  | TCGA-MN-A4N4 | 0.31391781 | Low risk |
| TCGA-05-4424 | 1.789303376 | High risk |  | TCGA-L4-A4E6 | 0.949683813 | Low risk |
| TCGA-38-4627 | 3.042507437 | High risk |  | TCGA-78-7536 | 1.285077767 | High risk |
| TCGA-44-5645 | 0.758866018 | Low risk |  | TCGA-05-4389 | 2.76198784 | High risk |
| TCGA-69-7764 | 0.980356945 | High risk |  | TCGA-97-7547 | 0.877634535 | Low risk |
| TCGA-55-8620 | 0.777216163 | Low risk |  | TCGA-62-8394 | 0.809533688 | Low risk |
| TCGA-55-A492 | 0.373494847 | Low risk |  | TCGA-38-7271 | 1.558349221 | High risk |
| TCGA-78-7220 | 5.787361098 | High risk |  | TCGA-49-AARQ | 0.60956373 | Low risk |
| TCGA-49-AARN | 0.815613899 | Low risk |  | TCGA-64-1678 | 1.792640008 | High risk |
| TCGA-50-8457 | 0.389290553 | Low risk |  | TCGA-44-A479 | 0.490089348 | Low risk |
| TCGA-91-8496 | 0.517686963 | Low risk |  | TCGA-97-7938 | 0.19369866 | Low risk |
| TCGA-55-8092 | 5.074677732 | High risk |  | TCGA-69-7974 | 0.909591369 | Low risk |
| TCGA-NJ-A4YQ | 1.518233424 | High risk |  | TCGA-44-3917 | 0.784180833 | Low risk |
| TCGA-69-8255 | 0.849999277 | Low risk |  | TCGA-05-4415 | 6.550874695 | High risk |
| TCGA-78-8640 | 2.013536985 | High risk |  | TCGA-78-7150 | 5.478676462 | High risk |
| TCGA-97-8174 | 0.561846672 | Low risk |  | TCGA-69-7763 | 0.407101001 | Low risk |
| TCGA-55-6987 | 0.915949763 | Low risk |  | TCGA-95-7947 | 1.347924137 | High risk |
| TCGA-49-6744 | 1.677887655 | High risk |  | TCGA-86-7711 | 0.78829928 | Low risk |
| TCGA-J2-8192 | 1.717402777 | High risk |  | TCGA-55-8621 | 0.753887844 | Low risk |
| TCGA-44-7671 | 0.896740225 | Low risk |  | TCGA-L9-A8F4 | 0.262079705 | Low risk |
| TCGA-49-AAR9 | 15.13516774 | High risk |  | TCGA-86-7953 | 1.493241415 | High risk |
| TCGA-80-5611 | 0.242650561 | Low risk |  | TCGA-95-7039 | 0.60934787 | Low risk |
| TCGA-71-6725 | 1.44518679 | High risk |  | TCGA-55-8206 | 0.498243561 | Low risk |
| TCGA-44-2656 | 1.683119797 | High risk |  | TCGA-44-7661 | 2.91144144 | High risk |
| TCGA-05-5715 | 2.622767691 | High risk |  | TCGA-J2-A4AD | 0.676688451 | Low risk |
| TCGA-64-5815 | 1.097062448 | High risk |  | TCGA-75-6206 | 1.187831889 | High risk |
| TCGA-55-7816 | 1.053414272 | High risk |  | TCGA-55-8616 | 1.612205096 | High risk |
| TCGA-99-8033 | 2.31878274 | High risk |  | TCGA-97-7552 | 1.345420728 | High risk |
| TCGA-73-4668 | 1.123663367 | High risk |  | TCGA-44-2657 | 0.396977591 | Low risk |
| TCGA-44-5644 | 0.467024882 | Low risk |  | TCGA-91-6840 | 2.304860908 | High risk |
| TCGA-38-4625 | 1.219059171 | High risk |  | TCGA-55-7907 | 1.578479808 | High risk |
| TCGA-86-6851 | 1.147507292 | High risk |  | TCGA-49-4510 | 0.254314311 | Low risk |
| TCGA-44-6779 | 3.892100994 | High risk |  | TCGA-55-7726 | 8.396340529 | High risk |
| TCGA-78-7537 | 0.89118962 | Low risk |  | TCGA-L9-A443 | 0.259649193 | Low risk |
| TCGA-67-3771 | 0.520330545 | Low risk |  | TCGA-53-7813 | 0.316890339 | Low risk |
| TCGA-55-8299 | 1.396701106 | High risk |  | TCGA-55-6970 | 2.064363929 | High risk |
| TCGA-44-A47A | 0.840994712 | Low risk |  | TCGA-99-7458 | 0.334336997 | Low risk |
| TCGA-05-4402 | 0.992668707 | High risk |  | TCGA-86-6562 | 1.607214667 | High risk |
| TCGA-49-4490 | 1.489930655 | High risk |  | TCGA-05-4398 | 0.945638912 | Low risk |
| TCGA-55-8090 | 1.144970896 | High risk |  | TCGA-78-7161 | 0.809067248 | Low risk |
| TCGA-99-AA5R | 0.496832301 | Low risk |  | TCGA-55-8087 | 0.345001147 | Low risk |
| TCGA-49-4486 | 0.391472482 | Low risk |  | TCGA-67-6216 | 0.346342876 | Low risk |
| TCGA-91-6847 | 3.712625309 | High risk |  | TCGA-64-1676 | 0.421099717 | Low risk |
| TCGA-50-5049 | 1.272490692 | High risk |  | TCGA-78-7160 | 0.540932174 | Low risk |
| TCGA-50-5066 | 0.727538145 | Low risk |  | TCGA-55-1595 | 0.313750196 | Low risk |
| TCGA-55-7727 | 0.892786438 | Low risk |  | TCGA-62-8402 | 0.48794345 | Low risk |
| TCGA-86-8674 | 0.155452839 | Low risk |  | TCGA-78-8648 | 1.114799689 | High risk |
| TCGA-69-A59K | 0.920164628 | Low risk |  | TCGA-55-A57B | 0.356143008 | Low risk |
| TCGA-44-2666 | 1.153653545 | High risk |  | TCGA-55-7903 | 0.669347433 | Low risk |
| TCGA-44-A47G | 0.783620257 | Low risk |  | TCGA-55-A493 | 2.123236225 | High risk |
| TCGA-55-8507 | 0.426047819 | Low risk |  | TCGA-55-A48X | 1.534205121 | High risk |
| TCGA-05-4433 | 1.802148699 | High risk |  | TCGA-05-4384 | 1.374295049 | High risk |
| TCGA-55-A4DG | 0.507338675 | Low risk |  | TCGA-97-A4M1 | 0.502217305 | Low risk |
| TCGA-49-4494 | 4.527774869 | High risk |  | TCGA-95-7567 | 1.219966615 | High risk |
| TCGA-44-6147 | 1.056379445 | High risk |  | TCGA-55-8302 | 1.996627782 | High risk |
| TCGA-49-AARO | 0.932256831 | Low risk |  | TCGA-75-7027 | 7.944329484 | High risk |
| TCGA-91-6848 | 22.62085344 | High risk |  | TCGA-49-4505 | 0.863057757 | Low risk |
| TCGA-50-8460 | 0.66430391 | Low risk |  | TCGA-44-6775 | 1.440885347 | High risk |
| TCGA-50-6673 | 1.228479621 | High risk |  | TCGA-44-2665 | 1.951562817 | High risk |
| TCGA-55-6642 | 1.454433943 | High risk |  | TCGA-55-A48Z | 0.805466016 | Low risk |
| TCGA-50-6591 | 1.992680383 | High risk |  | TCGA-44-3396 | 0.819910928 | Low risk |
| TCGA-78-7166 | 1.019957259 | High risk |  | TCGA-86-8281 | 0.126090248 | Low risk |
| TCGA-73-4676 | 0.881732656 | Low risk |  | TCGA-62-8397 | 0.795276213 | Low risk |
| TCGA-78-7148 | 3.07995466 | High risk |  | TCGA-78-7153 | 0.712453863 | Low risk |
| TCGA-93-7347 | 0.958143232 | High risk |  | TCGA-MP-A4T8 | 1.390369768 | High risk |
| TCGA-44-7672 | 1.978271234 | High risk |  | TCGA-44-7670 | 0.656407907 | Low risk |
| TCGA-55-7227 | 1.001545941 | High risk |  | TCGA-55-8512 | 0.237657215 | Low risk |
| TCGA-MP-A4T6 | 0.154346387 | Low risk |  | TCGA-50-5946 | 0.702660052 | Low risk |
| TCGA-05-4403 | 1.430437665 | High risk |  | TCGA-38-4631 | 6.345181 | High risk |
| TCGA-55-8511 | 0.984568861 | High risk |  | TCGA-73-7499 | 2.350681766 | High risk |
| TCGA-78-7167 | 3.933678568 | High risk |  | TCGA-55-7283 | 1.098291476 | High risk |
| TCGA-97-A4M0 | 0.444047596 | Low risk |  | TCGA-38-4626 | 0.474197257 | Low risk |
| TCGA-86-7714 | 0.885151007 | Low risk |  | TCGA-95-7562 | 2.36645911 | High risk |
| TCGA-86-8669 | 1.934811346 | High risk |  | TCGA-69-7760 | 2.201556148 | High risk |
| TCGA-78-7145 | 1.996949566 | High risk |  | TCGA-50-5935 | 0.645961745 | Low risk |
| TCGA-86-8075 | 0.903134355 | Low risk |  | TCGA-95-7043 | 0.978474436 | High risk |
| TCGA-86-A456 | 0.24292327 | Low risk |  | TCGA-55-6971 | 2.589350728 | High risk |
| TCGA-MP-A4SW | 0.30987958 | Low risk |  | TCGA-95-A4VN | 1.293404107 | High risk |
| TCGA-69-7973 | 7.368713963 | High risk |  | TCGA-J2-8194 | 0.572865337 | Low risk |
| TCGA-86-8671 | 0.755501143 | Low risk |  | TCGA-44-5643 | 2.14141348 | High risk |
| TCGA-35-4123 | 1.848422428 | High risk |  | TCGA-44-A4SS | 4.451061701 | High risk |
| TCGA-49-6761 | 0.912619853 | Low risk |  | TCGA-97-8171 | 2.254055941 | High risk |
| TCGA-44-A4SU | 0.759612685 | Low risk |  | TCGA-93-A4JP | 0.631962921 | Low risk |
| TCGA-86-8279 | 0.665585091 | Low risk |  | TCGA-50-5930 | 1.846184199 | High risk |
| TCGA-86-8358 | 1.197034918 | High risk |  | TCGA-64-5781 | 2.914780694 | High risk |
| TCGA-78-7162 | 1.657837513 | High risk |  | TCGA-69-8254 | 1.014347499 | High risk |
| TCGA-44-6777 | 1.072287483 | High risk |  | TCGA-50-7109 | 0.836497594 | Low risk |
| TCGA-05-4422 | 1.115896168 | High risk |  | TCGA-05-5428 | 1.448466191 | High risk |
| TCGA-49-4506 | 11.83099461 | High risk |  | TCGA-NJ-A4YP | 1.178569109 | High risk |
| TCGA-05-4417 | 0.885364798 | Low risk |  | TCGA-44-8117 | 0.477350801 | Low risk |
| TCGA-75-5146 | 0.71147609 | Low risk |  | TCGA-05-4425 | 1.326747651 | High risk |
| TCGA-86-8074 | 1.243806138 | High risk |  | TCGA-55-1596 | 1.39703013 | High risk |
| TCGA-97-7937 | 0.17060391 | Low risk |  | TCGA-91-6836 | 1.232198259 | High risk |
| TCGA-97-A4M7 | 0.464971668 | Low risk |  | TCGA-99-8032 | 0.905087177 | Low risk |
| TCGA-05-4420 | 1.542065346 | High risk |  | TCGA-44-7662 | 0.884370889 | Low risk |
| TCGA-78-7163 | 0.27649761 | Low risk |  | TCGA-44-2655 | 0.686656917 | Low risk |
| TCGA-86-7701 | 2.088965334 | High risk |  | TCGA-MP-A4TF | 80.68835495 | High risk |
| TCGA-67-3770 | 0.917843415 | Low risk |  | TCGA-67-6215 | 0.580252731 | Low risk |
| TCGA-38-4629 | 3.10908434 | High risk |  | TCGA-55-7911 | 0.738546967 | Low risk |
| TCGA-MP-A4TA | 5.992050762 | High risk |  | TCGA-75-6214 | 5.740113698 | High risk |
| TCGA-50-5931 | 0.670459128 | Low risk |  | TCGA-73-4677 | 0.844138399 | Low risk |
| TCGA-55-7994 | 1.503576737 | High risk |  | TCGA-97-7554 | 0.91136609 | Low risk |
| TCGA-55-7725 | 0.177138552 | Low risk |  | TCGA-05-4426 | 0.834893226 | Low risk |
| TCGA-MP-A4SY | 2.503577572 | High risk |  | TCGA-05-4382 | 0.508321347 | Low risk |
| TCGA-78-7154 | 2.762392315 | High risk |  | TCGA-J2-A4AE | 0.184731142 | Low risk |
| TCGA-73-4659 | 0.910718063 | Low risk |  | TCGA-50-6594 | 3.210185698 | High risk |
| TCGA-50-5068 | 0.655158459 | Low risk |  | TCGA-55-8505 | 2.247712654 | High risk |
| TCGA-05-4250 | 1.800789562 | High risk |  | TCGA-75-5125 | 0.511260552 | Low risk |
| TCGA-44-6776 | 1.335154997 | High risk |  | TCGA-62-A46V | 0.427911421 | Low risk |
| TCGA-05-4418 | 3.92747995 | High risk |  | TCGA-91-6829 | 1.223892675 | High risk |
| TCGA-55-7995 | 0.896567366 | Low risk |  |  |  |  |
