# Supplementary material for: Prognostic value and immune landscapes of anoikis-associated lncRNAs in lung adenocarcinoma
Source: Aging (Albany NY). 2024 Feb 5;16(3):2273–98. doi: 10.18632/aging.205481 (PMC10911388; doi:10.18632/aging.205481)
Supplement: Supplementary Tables 7 and 8 [file aging-16-205481-s006.pdf]

Supplementary Table 7. The 6 sensitive drugs in the low-risk group were obtained by chemotherapy drug sensitivity analysis at P<0.001.

Drug	P-value	L.median (25%,75%)	H.median (25%,75%)
**IGF1R signaling**			
BMS-754807_2171	<0.001	1.4(0.46-2.80)	2.29(0.87-4.24)
**JNK and p38 signaling**			
Doramapimod_1042	<0.001	83.61(69.75-104.75)	102.35(79.12-125.55)
**Mitosis**			
ZM447439_1050	<0.001	16.88(14.01-22.23)	19.95(15.60-27.00)
SB505124_1194	<0.001	9.81(7.58-11.49)	10.55(8.91-12.46)
**PI3K/MTOR signaling**			
PF-4708671_1129	<0.001	44.12(35.94-56.42)	52.27(40.16-67.21)
**RTK signaling**			
Axitinib_1021	<0.001	19.84(15.40-25.53)	24.51(19.14-31.54)

Abbreviation: H, High risk group; L, Low risk group.

Supplementary Table 8. The 59 sensitive drugs in the high-risk group were obtained by chemotherapy drug sensitivity analysis at P<0.001.

Drug	P-value	L.median (25%,75%)	H.median (25%,75%)
**Apoptosis regulation**			
Wee1 Inhibitor_1046	<0.001	7.47(3.92-17.87)	5.43(2.84-9.77)
PD0325901_1060	<0.001	1.81(1.13-3.32)	1.32(0.75-2.20)
IAP_5620_1428	0.001	166.18(101.98-300.14)	132.62(73.47-237.21)
Vinorelbine_2048	<0.001	0.04(0.02-0.16)	0.02(0.01-0.08)
**Apoptosis regulation**			
Palbociclib_1054	0.001	43.51(27.37-73.12)	34.73(21.48-54.85)
BI-2536_1086	0.001	1.52(0.97-2.46)	1.25(0.63-2.04)
MK-1775_1179	<0.001	1.81(0.98-4.43)	1.03(0.60-2.06)
VE821_2111	<0.001	60.1(34.42-139.30)	36.68(21.63-75.00)
**Chromatin other**			
MK-8776_2046	<0.001	24.11(12.15-59.18)	12.82(7.41-25.27)
**DNA replication**			
Gemcitabine_1190	0.001	0.6(0.21-1.62)	0.37(0.14-1.06)
Epirubicin_1511	0.001	0.36(0.20-0.76)	0.27(0.16-0.52)
Foretinib_2040	<0.001	2.61(1.44-4.64)	1.68(0.96-2.93)
Pyridostatin_2044	0.001	25.58(15.86-47.59)	20.14(13.58-35.02)
**EGFR signaling**			
Gefitinib_1010	<0.001	23.98(14.81-43.06)	17.29(11.02-32.56)
Erlotinib_1168	<0.001	13.92(9.28-22.93)	10.35(6.73-17.11)
Lapatinib_1558	0.001	19.14(9.89-42.41)	13.51(7.37-29.66)
AZD3759_1915	<0.001	13.02(8.81-23.27)	10.95(7.05-17.85)
Ulixertinib_2047	<0.001	9.95(5.74-16.58)	8.51(5.34-12.73)
**ERK MAPK signaling**			
Alisertib_1051	0.001	6.34(2.64-20.79)	4.51(1.84-10.41)
Trametinib_1372	<0.001	2.18(1.07-4.93)	1.23(0.52-3.19)
VE-822_1613	<0.001	30.55(14.81-75.84)	18.45(9.74-44.10)
ERK_6604_1714	<0.001	36.5(22.63-62.14)	28.49(15.34-48.95)
AZD4547_1786	<0.001	18(10.57-33.60)	13.67(8.02-21.89)
Buparlisib_1873	<0.001	2.47(1.77-4.16)	1.96(1.38-3.24)
**Genome integrity**			
Vinblastine_1004	<0.001	0.02(0.01-0.08)	0.02(0.01-0.04)
Talazoparib_1259	<0.001	28.19(15.37-52.10)	18.24(9.69-33.86)
Pevonedistat_1529	<0.001	2.1(1.02-5.31)	1.4(0.58-2.92)
**Hormone-related**			
Fulvestrant_1200	<0.001	16.13(10.95-29.25)	12.87(9.41-19.21)
GDC0810_1925	<0.001	129.24(93.10-205.90)	98.57(71.36-156.54)
**IGF1R signaling**			
AZD7762_1022	<0.001	1.03(0.58-2.41)	0.67(0.38-1.23)
Staurosporine_1034	<0.001	0.05(0.03-0.11)	0.03(0.02-0.06)
**Metabolism**			
GSK2606414_1618	0.001	38.55(24.86-64.48)	32.2(22.40-46.59)
**Mitosis**			
Cisplatin_1005	<0.001	30.97(14.04-72.36)	14.26(5.53-34.88)
Docetaxel_1007	<0.001	0.01(0.01-0.03)	0.01(0.00-0.01)
Luminespib_1559	<0.001	0.1(0.05-0.27)	0.07(0.04-0.14)
Savolitinib_1936	<0.001	14.36(7.64-26.69)	8.08(4.85-13.45)

**p53 pathway**			
MIRA-1_1931	<0.001	196.28(107.82-367.99)	147.05(94.10-247.43)
**PI3K/MTOR signaling**			
Dactolisib_1057	<0.001	0.19(0.11-0.37)	0.14(0.08-0.26)
GNE-317_1926	<0.001	1.74(0.99-3.40)	1.17(0.76-2.24)
Ipatasertib_1924	0.001	27.69(14.41-62.63)	21.89(11.05-40.65)
LJI308_2107	0.001	166.78(102.37-296.89)	124.37(77.98-205.04)
Pictilisib_1058	<0.001	3.92(2.19-8.61)	2.71(1.59-5.31)
Taselisib_1561	<0.001	9.15(4.13-19.07)	5.97(2.48-14.13)
Paclitaxel_1080	<0.001	0.07(0.03-0.24)	0.04(0.02-0.10)
YK-4-279_1239	<0.001	9.56(4.99-26.66)	6.15(3.26-14.90)
Alpelisib_1560	<0.001	36.02(17.94-77.46)	22.27(10.47-47.92)
AMG-319_2045	<0.001	115.83(75.92-196.16)	92.09(61.12-149.61)
AT13148_2170	0.001	30.38(17.05-72.24)	21.85(12.17-55.76)
**Protein stability and degradation**			
BMS-536924_1091	<0.001	9.14(5.90-13.51)	6.47(4.29-10.45)
BPD-00008900_1998	<0.001	91.93(63.06-146.75)	69.9(47.88-112.14)
**RTK signaling**			
5-Fluorouracil_1073	<0.001	123.65(55.68-338.28)	63.96(25.47-183.49)
**Other**			
Cytarabine_1006	<0.001	6.77(3.12-13.02)	4.26(1.83-8.59)
Dasatinib_1079	<0.001	5.49(2.05-17.88)	3.47(0.70-11.36)
Dabrafenib_1373	0.001	101.23(54.01-186.31)	74.25(34.06-145.65)
Temozolomide_1375	<0.001	328.03(226.50-763.95)	274.93(162.41-466.20)
I-BET-762_1624	<0.001	29.3(19.04-47.07)	21.97(14.31-37.35)
AZD6738_1917	<0.001	8.84(4.63-20.71)	5.38(2.63-9.13)
VX-11e_2096	<0.001	18.18(12.24-34.21)	12.77(7.66-21.69)
**Unclassified**			
BDP-00009066_1866	<0.001	9.61(7.16-17.15)	8.07(5.66-11.79)

Abbreviation: H, High risk group; L, Low risk group.
